# Comparative Effectiveness of Individual, Group, Family and Combined Therapy Formats on Suicidality: A Meta‐Analysis

**DOI:** 10.1002/cpp.70112

**Published:** 2025-07-02

**Authors:** Nikki Lyka van Eijk, Renske Gilissen, Daan Creemers, Lizanne J. S. Schweren, Wouter van Ballegooijen

**Affiliations:** ^1^ 113 Suicide Prevention Amsterdam the Netherlands; ^2^ Leiden University Leiden the Netherlands; ^3^ Radboud University Nijmegen the Netherlands; ^4^ GGZ Oost‐Brabant Boekel the Netherlands; ^5^ Vrije Universiteit Amsterdam Amsterdam the Netherlands; ^6^ Amsterdam University Medical Centre (location VUMC) Amsterdam the Netherlands

**Keywords:** family therapy, group therapy, meta‐analysis, psychotherapy, suicide

## Abstract

Through carefully choosing psychotherapeutic interventions based on treatment characteristics, we may increase the relatively small effect sizes for suicidal outcomes. This study aims to evaluate the efficacy of different psychotherapy formats in reducing suicidal ideation and attempts, including individual, group and family‐based therapies, as well as combined modalities involving individual and group or individual and family interventions. We conducted a meta‐analysis after systematically searching databases for randomised controlled trials up to April 1, 2024. Included studies targeted any mental health issue, were delivered in any setting, compared with any control group, and reported on suicidal ideation or attempts. Data extraction and quality assessment were performed independently by two researchers, and a mixed‐effects model was used for data synthesis. One hundred and seventy‐two studies were eligible, totalling 198 comparisons with a total of 22,440 participants. Individual therapy significantly reduced suicidal ideation (*g* = −0.33, *p* < 0.001, *k* = 72 comparisons), as did group therapy (*g* = −0.39, *p* < 0.001, *k* = 27 comparisons). For reducing suicide attempts, individual therapy (RR = 0.75, *p* < 0.001, *k* = 63 comparisons), and notably, combinations of individual with group (RR = 0.42, *p* = 0.010, *k* = 7 comparisons) and individual with family‐based therapy (RR = 0.59, *p* = 0.041, *k* = 8 comparisons) were effective. Subgroup analysis showed that combined therapies were associated with larger effect sizes compared to single modalities in reducing suicide attempts (*p* < 0.001). This meta‐analysis highlights that combined psychotherapy approaches, integrating individual sessions with group or family sessions, yields significantly higher effect sizes, reducing the risk of suicide attempts by 50%. These findings support the adoption of combined therapeutic strategies in clinical settings to effectively address suicidality.

**Summary:**

- Individual or group therapy is effective for reducing suicidal ideation. Family therapy seems less effective than other formats.
- Individual therapy, or combining individual therapy with group therapy or family therapy, is effective for preventing suicide attempts.
- A combination of individual therapy with group or family therapy seems more effective at preventing suicide attempts than other formats.

## 1 Introduction

Suicide is an important cause of death in most mental disorders (Chesney et al. 2014), and suicidal ideation and suicide attempts are risk factors for future deaths by suicide (Hubers et al. 2018; Large et al. 2021). Previous studies have shown that various psychotherapies are effective in reducing suicidal ideation or preventing suicide attempts (Calati and Courtet 2016; Fox et al. 2020; Tarrier et al. 2008). Many studies also show that various types of psychotherapy are similarly effective (Barth et al. 2016). Interventions such as cognitive behavioural therapy (CBT), dialectical behaviour therapy (DBT) problem‐solving therapy, and others have all been found effective, though effect sizes tend to be limited (DeCou et al. 2019; Nordentoft et al. 2005; Witt et al. 2021). A study comparing treatment manuals from five treatments for suicidality found many similarities in strategies, which could explain their similar outcomes (Weinberg et al. 2010). Moreover, interventions tend to be effective for suicidal ideation or suicide attempts, but not both. Therefore, it is important to look at both outcomes separately, as distinct processes and risk factors may underlie the development of suicidal ideation and the progression from ideation to action according to modern theories of suicidal behaviour (Klonsky et al. 2021; O'Connor and Kirtley 2018). However, a study assessing the potential mediating effect of three core components of the interpersonal theory for suicide found these were unrelated to treatment success (Bryan et al. 2018), suggesting that mechanisms underlying the effect of psychotherapy for suicidality may lie elsewhere. Some suggest this is because theoretical models of suicide and treatment models are not well‐aligned, with non‐specific treatment factors playing an important role as well (Michel 2021).

Apart from varying in content, psychotherapeutic interventions can differ in their format, ranging from individual therapy, group therapy, family‐based treatment, to combinations of different formats (Bieling et al. 2022; Mohr et al. 2012). It is unclear whether some formats are more effective than others in treating suicidal ideation or attempts. Format could potentially impact non‐specific factors such as connectedness (Aherne et al. 2018). A scoping review on group treatments for suicidal individuals found that group interventions are effective in reducing suicidal thoughts and behaviours (Sullivan et al. 2021). Moreover, the role of the family in suicide prevention has gained considerable attention in the last decades (Prabhu et al. 2010). A network meta‐analysis found that family‐based treatment was associated with lower suicidal ideation in children and adolescents (Bahji et al. 2021). Various ways of involving the family in treatment have been suggested, with some studies saying it may prevent an early dropout from treatment (Gorman et al. 2023).

A combination of individual therapy with some other format is sometimes offered, for example in DBT (Linehan et al. 2006, 2015). Oftentimes this treatment is relatively intense, with several sessions per week. A meta‐regression on depression treatment found that two sessions per week increased the effect size with *g =* 0.45 compared to one session per week, suggesting that a higher concentration of sessions within a short timeframe may be more effective (Cuijpers et al. 2013). However, a review of psychotherapies targeting suicidality and depression for patients with borderline personality disorder found no difference in effect between more and less intensive therapies (Davidson and Tran 2014). It is therefore unclear what mechanism underlies effectivity, and whether the combination of individual therapy with another format, or the intensity of the treatment, are associated with better outcomes.

Conclusive evidence regarding the effectiveness of different formats of psychotherapy on reducing suicidal ideation and attempts is lacking. Therefore, the current study has two primary objectives. Firstly, we examined the effect of individual, group, and family‐based interventions, as well as combinations between individual and group or individual and family‐based interventions in treating suicidal ideation and suicide attempts. Secondly, we aimed to compare the aforementioned five formats to investigate which format yields the best outcomes for suicidality.

## 2 Method

### 2.1 Study Search and Selection

The full search strategy and search strings can be found on https://docs.metapsy.org/databases/suicide/. A systematic search of papers published up to 1 April 2024, was run in PubMed, Embase, PsycINFO, Web of Science, Scopus and The Cochrane Central Register of Controlled Trials. This study is part of a meta‐analytic research domain (MARD) aiming to investigate the effect of psychotherapy on suicide.

Trials were included in the database if they met the following criteria: (1) had a randomised design comparing two or more groups; (2) examined psychotherapy (the use of psychological methods to change behaviour or overcome problems) targeting any mental health problem and delivered in any setting (including guided digital treatments); (3) compared the intervention with a control group (such as care as usual, enhanced care as usual, or waiting list); (4) reported suicidal ideation, suicide attempts, death by suicide, or self‐harm leading to hospitalisation as primary or secondary outcome; (5) were written in English, Dutch, German or Greek. Studies were excluded if they: (1) employed an intervention that did not involve any psychotherapeutic technique, such as an automated text message or community intervention; (2) measured only suicidal ideation on a categorical scale, such as a single item of a questionnaire for depression; and (3) only reported suicide as an adverse event and no other suicide‐related outcome. We included studies on all age groups. All abstracts and full texts were screened by two independent researchers. Conflicts were discussed with a third researcher, as well as the decision to include or exclude a study based on the full texts.

For this meta‐analysis, we were interested in comparing treatment formats using subgroup analysis. Format was defined as the way therapy was delivered to the participant. Formats that did not have a sufficient number of studies were excluded from the analysis. Excluded formats were guided self‐help (3 studies) and school‐based (1 study).

### 2.2 Data Extraction

The extracted data and documentation for the database can be found at https://www.metapsy.org/suicide‐prevention. Data are derived from a living systematic review that is updated annually. Data were extracted by two independent researchers. Conflicts were solved by discussion and, if necessary, discussed with a third researcher.

In the present paper, we reported participant characteristics (age group, percentage of women, recruitment method, suicidality as inclusion criterion), characteristics of the treatment (type of therapy, treatment format, whether the treatment directly targeted suicidality), and general characteristics (country where the study took place, type of control group).

### 2.3 Risk of Bias

Risk of bias of the included studies was assessed using the ‘Risk of bias’ (RoB) tool 2.0, developed by the Cochrane Collaboration. The RoB tool 2.0 assesses possible bias in randomised trials in the following domains: adequate randomisation and allocation, knowledge of the allocation among participants and those delivering the intervention, adequate handling of missing data, adequate measuring of the outcome, analysing and reporting the outcomes. Risk of bias assessment was conducted by two independent researchers, and disagreements were solved by discussion, and, if necessary, a third researcher.

### 2.4 Outcome Measures

We extracted two types of outcomes. The first was post‐treatment suicidal ideation (SI), which is usually presented as a mean and standard deviation on a self‐report scale or clinical interview. The second is the number of people who attempted suicide between the baseline and post‐treatment assessments. People who died by suicide or were hospitalised after a self‐harm episode were also considered to have attempted suicide. For the outcome SI, we calculated the effect size indicating the difference in SI severity between the two groups at post‐test (Hedges' *g*) for each comparison between a psychological treatment and a control condition. When the M, SD and N were not reported in the paper, we extracted change scores from baseline to post‐test, or binary outcomes (such as the proportion of participants who responded or remitted). If these data were also not reported, we used any other statistic indicating the difference between the two conditions at post‐test and that can be transformed to effect sizes (such as *p* value or *t* value). When data from intention‐to‐treat and per‐protocol analyses were available, we extracted the intention‐to‐treat outcomes. We also examined suicide attempts (SA), for which we calculated the relative risk (RR) as the proportion of people who attempted suicide in the psychotherapy group divided by the proportion in the control group. When a trial had multiple treatment or control groups, all groups of interest were extracted. These types of trials were distinguished as ‘multiarm’ trials in the dataset, so that these can be handled accordingly in the analyses (i.e., accounting for effect size dependency).

### 2.5 Data Analysis

To answer the first research question, we were interested in the effectiveness of each format. For this, we had a combination of two outcomes (SI and SA) and five formats (individual, group, family, individual and group, individual and family). Consequently, we conducted 10 meta‐analyses: one for each outcome for each format. Heterogeneity was based on the total estimate of $\tau^{2}$, which quantifies the variance of the true effect sizes. While $\tau^{2}$ is a measure capturing the true variance, which is not influenced by factors such as sample size, it can also be harder to interpret. Therefore, both $\tau^{2}$ and $I^{2}$ are reported. $\tau^{2}$ is interpreted on the same scale as our effect size metric, i.e., Hedges' g for SI and the RR for SA. Then, to answer the second research question, we conducted two new meta‐analyses on the entire dataset with a subgroup analysis for both outcomes to investigate any differences in effect sizes between the formats. A subgroup analysis is a post hoc procedure or moderator analysis aimed at studying characteristics that may influence the overall effect size. Similarly to a meta‐regression, it is a tool to investigate variation between studies. For all meta‐analyses, a three‐level model with correlated and hierarchical effects (CHE) was used. This is one of the more complex estimation models for a meta‐analysis, with effect sizes nested in studies and separate variance–covariance matrices for multi‐arm trials. This model tends to be a good approximation for data sets with unknown or complex dependency structures, and since results from multi‐arm trials cannot be independent, we believe this added complexity would improve our estimate of the pooled effect. To study the robustness of the effect, various sensitivity analyses were conducted, including models with only one effect size for multi‐arm trials, models where outliers were removed, and models where influential cases were removed. All sensitivity analyses can be found in Appendix A. Publication bias was assessed visually with forest plots and statistically using Eggers' test.

### 2.6 Transparency and Openness

This study is part of a larger meta‐analytic project examining the effect of psychotherapeutic interventions on suicidal ideation and suicide attempts(M. X. Hu et al. 2020) (Prospero registration number: CRD42020140573). This larger project is a MARD, which aims to capture a whole specific field by including multiple PICOS (PICO stands for Participants, Intervention, Comparator, Outcome) and being continually updated as a living systematic review (Cuijpers et al. 2022). As such, this MARD captures more than what can be covered by a single (network) meta‐analysis. The dataset used for the analyses in this paper can be found online at www.metapsy.org. The main analyses were conducted in RStudio with R version 4.3.1 (Posit team 2024; R Core Team 2023), using the metapsyTools package (Harrer and Kuper 2022). The metapsyTools package was specifically developed for the MARDs of Metapsy (metapsy.org). Descriptive analyses were conducted using Jamovi (The Jamovi Project 2023). The R code used to produce these results can be found in Appendix E.

## 3 Results

**FIGURE 1:**
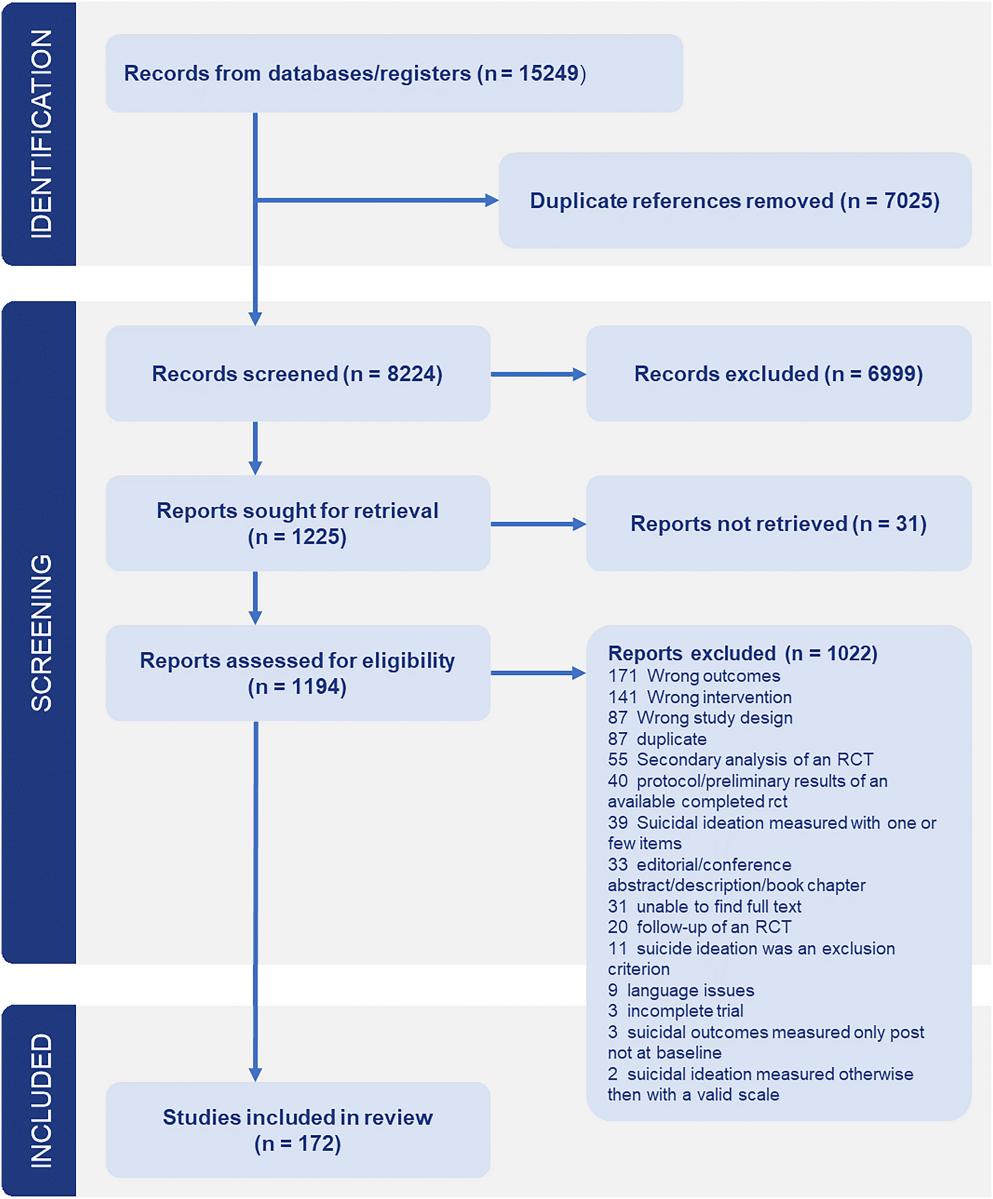
PRISMA flow diagram.

After screening a total of 15,249 abstracts (8224 after removal of duplicates), we retrieved 1194 full‐text papers for consideration. After the full‐text screening, a total of 172 studies met the inclusion criteria with a total of 22,440 participants. A list with the references for each included study can be found in Appendix B. From these 172 studies, a total of 198 comparisons were extracted. The PRISMA flow chart describing the inclusion process is presented in Figure 1. In total, 91 studies were included that investigated individual therapy (54.2%), 36 studies investigated group therapy (21.4%), 15 studies investigated family‐based treatment (8.9%), 11 studies investigated a combination of individual and group treatment (6.5%) and 15 studies investigated a combination of individual and family‐based treatment (8.9%). Selected characteristics of the included studies are summarised by format in Table 1. A full overview of characteristics per study is presented in Appendix C. Studies were predominantly from western high‐income countries across all formats, recruited largely from clinical samples and had a high percentage of women. A variety of different interventions was offered, with cognitive behavioural therapy, dialectical behavioural therapy and family‐based therapy among the most prevalent. Most studies employed care as usual as their control group. The family‐based studies and those employing both individual and family‐based treatment had younger samples than studies investigating individual and/or group therapies, where most participants were adults.

**TABLE 1:** Summarised characteristics of included studies.

Characteristic		Format				
		Individual	Group	Family‐based	Individual and group	Individual and family‐based
Total number of studies		91	36	15	11	15
Total number of participants		12,382	5071	2310	925	1752
Recruitment	Clinical	61.1%	58.8%	66.7%	81.8%	85.7%
	Community	11.1%	26.5%	0%	9.1%	0%
	Other	27.8%	14.7%	33.3%	9.1%	14.3%
Age group	Child	0%	2.8%	13.3%	0%	6.7%
	Adolescent	11.2%	13.9%	73.3%	0%	80%
	Young adult	10.1%	8.3%	0%	18.2%	0%
	Adult	78.6%	75%	13.3%	81.8%	13.3%
Average perc. women		58.6%	65.7%	68.2%	63.1%	74%
Suicidality as inclusion criterion	Yes	68.1%	44.4%	66.7%	72.7%	53.3%
	No	31.9%	55.6%	33.3%	27.3%	46.7%
Intervention targeting suicidality directly	Yes	60.2%	38.7%	73.3%	90.9%	58.3%
	No	39.8%	61.3%	26.7%	9.1%	41.7%
Intervention	Assip	3.3%	0%	0%	0%	0%
	Cams	4.4%	2.8%	0%	0%	0%
	Cbt	37.4%	36.1%	13.3%	9.1%	33.3%
	Dbt	2.2%	2.8%	0%	45.5%	20%
	Dyn	7.7%	0%	0%	9.1%	6.7%
	Fam	1.1%	0%	86.7%	0%	13.3%
	Img	7.7%	0%	0%	0%	0%
	Ipt	1.1%	2.8%	0%	0%	0%
	Mbct	0%	5.6%	0%	9.1%	0%
	Mbt	0%	0%	0%	9.1%	6.7%
	Mea	1.1%	0%	0%	0%	0%
	Mixed	2.2%	0%	0%	0%	0%
	Pst	9.9%	5.6%	0%	0%	6.7%
	Spi	1.1%	0%	0%	0%	0%
	Other	20.9%	44.4%	0%	18.2%	13.3%
Control	Cau	64.8%	44.4%	33.3%	54.5%	53.3%
	Ecau	8.8%	13.9%	33.3%	27.3%	20%
	Placebo	1.1%	0%	0%	0%	0%
	Supp	5.5%	0%	13.3%	0%	13.3%
	Wl	11%	22.2%	6.7%	9.1%	6.7%
	Other ctr	8.8%	19.4%	13.3%	9.1%	6.7%
Country	AU	7.7%	13.9%	0%	9.1%	6.7%
	CAN	0%	2.8%	0%	0%	0%
	EAS	3.3%	11.1%	0%	9.1%	0%
	EU	18.7%	27.8%	13.3%	9.1%	0%
	UK	16.5%	11.1%	13.3%	27.3%	6.7%
	USA	36.3%	25%	73.3%	45.5%	73.4%
	Other	17.6%	11.1%	0%	0%	13.3%
RoB	Low	3.8%	3%	6.7%	0%	0%
	Some concerns	7.5%	0%	6.7%	12.5%	0%
	High	88.8%	97%	76.7%	87.5%	100%

Abbreviations: AU, Australia or New Zealand; Assip, Attempted Suicide Short Intervention Program; CAMS, Collaborative Assessment & Management of Suicidality; CAN, Canada; CAU, care as usual; CBT, cognitive behavioural therapy; DBT, dialectical behavioural therapy; DYN, psychodynamic therapy; EAS, East Asia; ECAU, enhanced care as usual; EU, European Union; FAM, family‐based therapy; IMG, EMDR and imagery‐based therapies; IPT, interpersonal therapy; MBCT, mindfulness‐based cognitive therapy; MBT, mentalisation‐based Therapy; MEA, meaning making therapy; PST, problem‐solving therapy; RoB, risk of bias; SPI, safety‐planning or related interventions; SUPP, supportive counselling; UK, United Kingdom; USA, United States of America; WL, wait‐list.

This series of meta‐analyses systematically examined the effect of therapeutic interventions on suicidal ideation and suicide attempts. A three‐level meta‐analysis model with correlated and hierarchical effects was used to estimate the models. These findings are summarised in Table 2 for suicidal ideation and Table 3 for suicide attempts. Individual interventions showed a small to medium effect in reducing SI at post‐treatment (*g* = −0.33, 95% CI [−0.46; −0.19]), as well as in reducing SA (RR = 0.75, 95% CI [0.65;0.87]). This means that the difference in suicidal ideation was approximately a third of a standard deviation between the intervention and the control group, and there was a 25% reduction in suicide attempts in the intervention group compared to the control group. This effect was robust, showing a similar effect size and *p* value across various model estimations (all model estimations for each format are reported in Appendix A). Heterogeneity was much higher for studies reporting on SI compared to SA. Solely group interventions showed a small to medium effect for SI as well (*g* = −0.39, 95% CI [−0.46; −0.18]). This effect was also robust. However, these group interventions were not effective in reducing SA, with a RR of 0.92 (95% CI [0.4;2.15]). Solely family‐based interventions were not effective in reducing SI (*g* = −0.15, 95% CI [−0.34;0.04]), nor in reducing SA (RR = 0.81, 95% CI [0.44;1.5]). A combination of individual and group sessions showed a small to moderate effect for SI, albeit not statistically significant (*g* = −0.33, 95% CI [−0.69;0.03]). The effect on SA was much larger, with a RR of 0.42 for combined individual and group interventions (95% CI [0.27;0.67]). This indicates that the risk of suicidal behaviour was significantly reduced by 58% in the intervention condition. This effect was robust across all sensitivity analyses and had very low heterogeneity. Combining individual and family‐based interventions had the largest effect size for SI of any format (*g* = −0.43, 95% CI [−1.21;0.35]), which was however not statistically significant. This group of studies also had high heterogeneity. The effect on SA was statistically significant and moderate in size (RR = 0.59, 95% CI [0.36;0.96]), indicating that the risk of suicide attempts in the intervention group was reduced by 41%. This effect was also robust in all model estimations. Forest plots for each format can be found in Appendix D.

**TABLE 2:** Meta‐analysis results per format for suicidal ideation.

Format	*k*	Effect size^a^	95% CI	*p*	Total $\tau^{2}$	$I^{2}$
Individual	72	−0.33	[−0.46;‐0.19]	**< 0.001**	0.188	77.8%
Group	27	−0.39	[−0.6;‐0.18]	**0.** **001**	0.206	82.4%
Family based	10	−0.15	[−0.34;0.04]	0.106	0.018	26.6%
Individual and group	7	−0.33	[−0.69;0.03]	0.063	0.074	57.6%
Individual and family based	12	−0.43	[−1.21;0.35]	0.231	0.712	97.8%

*Note:* Estimation based on Three‐level model (CHE).

^a^ Effect size for ideation is Hedges' *g*.

**TABLE 3:** Meta‐analysis results per format for suicide attempts.

Format	*k*	Effect size^a^	95% CI	*p*	Total $\tau^{2}$	$I^{2}$
Individual	63	0.75	[0.65;0.87]	**< 0.001**	0.052	23.6%
Group	11	0.92	[0.4;2.15]	0.673	0	0%
Family based	10	0.81	[0.44;1.5]	0.431	0.179	38.4%
Individual and group	7	0.42	[0.27;0.67]	**0.** **010**	0	0%
Individual and family based	8	0.59	[0.36;0.96]	**0.** **041**	0	0%

*Note:* Estimation based on three‐level model (CHE).

^a^ Effect size for attempts the relative risk.

A subgroup analysis was conducted for each outcome to investigate differences in effect between the five formats investigated above. As expected, effect sizes for each format on suicidal ideation were comparable to those in the individual meta‐analyses, while confidence intervals were similar or narrower. In the subgroup analysis, all studies were entered together, with format as a moderator of the effect size. There was a significant difference in effectiveness between the five formats (*p* < 0.001). This was mostly driven by family‐based interventions having a much smaller effect size than the other formats. The subgroup analysis on preventing suicide attempts also revealed a statistically significant difference between the formats (*p* < 0.001). The effect of each format mirrors the results from the individual meta‐analyses, showcasing a much larger effect for the two combined formats (individual and group/individual and family‐based) over any single format.

Risk of Bias was predominantly high across all five formats, with only a few studies receiving some concerns or low as rating. In most cases, this was the result of inadequate handling of missing data, or missing information with regard to protocols, the analysis, and the reporting of outcomes. Furthermore, the lack of blinding in most studies as well as using self‐report on most outcomes led to higher risk of bias scores.

**FIGURE 2:**
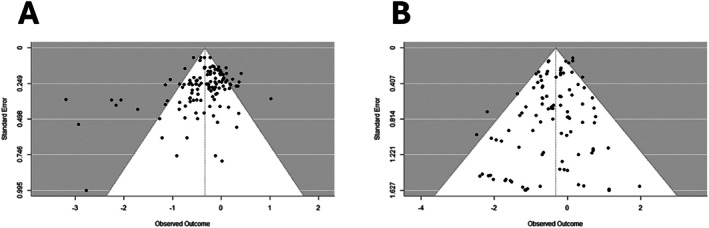
Funnel plots for suicidal ideation (A) and suicide attempts (B) based on the three‐level model with correlated effects.

To investigate whether publication bias may have affected the results, funnel plots were produced. These funnel plots are shown in Figure 2. The plot for suicidal ideation (plot A in Figure 2) suggests most studies have a relatively low standard error. To test the asymmetry in the plot, Eggers' test was used. This test was not available for the three‐level model and was instead conducted for the overall model, which did not indicate the presence of asymmetry (intercept = −0.44, *p* = 0.181). The funnel plot for suicide attempts looked similarly symmetrical (plot B in Figure 2), although Eggers' test suggested there was asymmetry (intercept = −0.51, *p* = 0.004).

## 4 Discussion

Various types of interventions have been shown to effectively reduce suicidal thoughts and/or attempts. In the present study, we looked at the effectiveness of five different formats of delivering psychotherapeutic interventions, and whether there were differences in effectiveness. Studies either targeted suicidality directly or indirectly through associated symptoms such as depression or anxiety. The results show that for reducing SI, solely individual therapy and solely group therapy were effective, though effect sizes were small. For preventing SA, individual therapy, individual and group therapy, and individual and family‐based therapy were effective. Providing a combination of family or group‐based sessions with individual sessions yielded a relative risk of 0.42–0.59, which was lower than those for any other format. Moreover, looking at a subgroup analysis, the results show that there was both a significant difference between formats in reducing suicidal ideation as well as for preventing suicide attempts.

Combining individual and group sessions is a strategy that has been advocated for in mental healthcare since the 1950s, with many arguments for why this combination should be adopted (Patterson et al. 1956; Rutan and Alonso 1982). This is in line with our results which show a 58% reduction in suicide attempts with this combined format. A recent study directly comparing combined individual and group therapy against predominantly group therapy for individuals with borderline personality disorder (BPD) revealed that the combined format was superior with regard to BPD severity as well as treatment retention (Arntz et al. 2022). A possible explanation for this may stem from the dual engagement approach. Research has highlighted connectedness as a protective factor against suicidality (Gill et al. 2023). A therapy model that blends one‐on‐one sessions with group interactions provides a unique supportive environment. Patients are offered a safe space to share sensitive thoughts and feelings, and a group setting where they can practise expressing these thoughts and foster social connection under supervision from a therapist. Moreover, therapists highlight that self‐disconnect may be at the core of suicidality, and that working to restore connectedness and interpersonal skills is a crucial strategy to prevent suicide (Aherne et al. 2018). This combination of the individual connection with the therapist and a group setting to enhance relational skills may be an effective mechanism.

Furthermore, combining individual sessions with family‐based treatment also yielded a 41% reduction in suicide attempts. The role of family in treating suicidal thoughts and behaviours is not to be underestimated, and active participation in treatment is not only beneficial to those experiencing suicidality, but also to their family (Grant et al. 2015). Furthermore, family members may provide additional information relevant to the treatment that could not be obtained from the patient (Edwards et al. 2021). However, contrary to previous research, solely family‐based treatment was not effective in our study. A possible explanation for this discrepancy is that we included twice as many studies as the previous meta‐analysis, as well as studies focused on adults (Bahji et al. 2021). Since a combination of family‐based treatment with individual treatment was effective, we see clear added value of the systemic approach. In fact, a review on family‐based treatment for adults found it to be effective for a range of psychological problems (Carr 2019). The fact we found no clear evidence for solely family‐based treatment in this study could be due to various factors, and more research is needed to explore the difference between the single and the combined format. Especially since the age distribution for family‐based treatment was the least similar to the other treatments, more research should focus on the effect of family‐based treatment in adults. Involving family and carers in care for suicide prevention is a key aspect of many international guidelines (American Psychiatric Association 2003; National Institute for Health and Care Excellence 2022; NVvP 2025; Sarkhel et al. 2023), but clinicians report the family approach is difficult in practice (Rothes et al. 2014). We hope this study motivates clinicians to increase involvement by showing the clear benefit compared to individual treatment only.

While a subgroup analysis is an effective way of investigating differences between groups in a meta‐analysis, a network meta‐analysis would have been a more direct and effective way to compare groups. However, there were insufficient studies directly comparing two or more formats to allow for a network meta‐analysis to be conducted. Because of this, other characteristics that differ between studies (e.g., recruitment method, age group, type of intervention) may have played a role that we could not control for. To combat this large opportunity for heterogeneity, we conducted a mixed‐effects model to pool the data. These models assume that studies do not only differ due to sampling error, but also because each study is seen as being from its own unique population, rather than all studies belonging to one population. A second limitation is that the subgroups were very different in size, making it difficult to compare. Yet even with limited studies, we were able to show a clear result on the effect of individual and group and individual and family‐based treatment combinations. A third limitation is that many studies only included results on suicidal ideation or attempts as a secondary outcome, and interventions often did not target suicidality directly. One review suggested that direct interventions targeting suicidality may be more effective than indirect interventions (Meerwijk et al. 2016). However, another meta‐analysis found no clear differences between direct and indirect interventions for improving suicidal outcomes (Van Ballegooijen et al. 2024). In our sample, combined individual and group interventions were more likely to target suicidality directly, and it is unclear whether the increased effectiveness is partly influenced by this. Lastly, while we investigated the effect of five different formats, there are many other formats that we were unable to investigate. Internet‐based interventions show promising results, as do telephone‐delivered interventions (Büscher et al. 2020; Comendador et al. 2023). As more research on diverse formats becomes available, more fine‐grained subgroup analyses can be conducted. Other characteristics influencing the effect could also be explored, such as the number of sessions, the length of the treatment, or age. In this study, we did not have enough comparisons to conduct further meta‐regression analyses to test these associations.

Future research on psychotherapy for suicidal thoughts and behaviours should focus on more head‐to‐head trials. Current network meta‐analyses show that comparisons can often only be made between active treatment conditions and treatment as usual, which limits their power (F.‐H. Hu et al. 2024; Jeong et al. 2023). Dismantling trials can also help us identify which components within psychotherapeutic interventions are most effective in reducing SI or preventing SA, which could also help shed light on why some interventions work better for SI or for SA. Another promising avenue for future research is the use of individual participant data to identify what treatment works best for whom. While this is more common in other mental healthcare fields, we have identified only one published individual participant data meta‐analysis studying psychotherapy with outcomes on suicidal ideation (Büscher et al. 2022).

This study is the first to meta‐analyse the effectiveness of group therapy, family‐based therapy, and a combination of different formats for suicidal thoughts and behaviours. Furthermore, it is also the first study to formally look at differences in effect sizes between different formats. Our results may aid clinicians in determining which treatment to offer when treating patients with suicidality. Since the results show that combining individual treatment with group or family therapy may reduce suicide attempts by 50% compared to only 25% when only individual therapy is delivered, this strengthens our beliefs that both next‐of‐kin and peers with lived experience contribute significantly to someone's health and well‐being.

## Author Contributions

Conceptualisation: N.L.E., R.G., D.C., L.J.S.S., W.B.; data curation: N.L.E., W.B.; formal analysis: N.L.E.; funding acquisition; investigation: N.L.E., W.B.; methodology: W.B.; project administration: N.L.E., L.J.S.S.; resources: R.G.; software; supervision: R.G., D.C., L.J.S.S., W.B.; validation: W.B.; visualisation: N.L.E.; writing – original draft: N.L.E.; writing – review and editing: N.L.E., R.G., D.C., L.J.S.S., W.B.

## Conflicts of Interest

The authors declare no conflicts of interest.

## Transparency Declaration

This manuscript is an honest and transparent account of the study being reported; no aspects of the study have been omitted from this manuscript.

## Analytic Code Availability

The analytic code for the analyses included in this manuscript can be found in the appendices.

## Research Material Availability

No additional research materials apart from those mentioned above were used for the current study.

## Supporting information


**Data S1** Supplementary information.

## Data Availability

Most of the data extracted for this meta‐analysis are publicly available at https://www.metapsy.org/suicide‐prevention. See https://docs.metapsy.org/databases/suicide/ for documentation. The complete dataset is available on request.
