# Supplementary material for: Comparative Effectiveness of Individual, Group, Family and Combined Therapy Formats on Suicidality: A Meta‐Analysis
Source: Clin Psychol Psychother. 2025 Jul 2;32(4):e70112. doi: 10.1002/cpp.70112 (PMC12217431; doi:10.1002/cpp.70112)
Supplement: Supplementary file 1 — Data S1 Supplementary information. [file CPP-32-e70112-s001.docx]

# Appendices

## Appendix A. Full results of each meta-analysis

| **Individual - Ideation** | ***k*** | ***g*** | **CI** | ***p*** | ***I*^2^** | **CI** | **PI** | **NNT** |
| --- | --- | --- | --- | --- | --- | --- | --- | --- |
| **Overall** | 72 | -0.30 | [-0.42; -0.18] | <0.001 | 67.77 | [58.89; 74.73] | [-1.09; 0.49] | 10.66 |
| **Combined^a^** | 64 | -0.30 | [-0.44; -0.17] | <0.001 | 70.24 | [61.68; 76.89] | [-1.17; 0.56] | 10.45 |
| **One ES/study (lowest)^b^** | 59 | -0.35 | [-0.5; -0.21] | <0.001 | 70.31 | [61.37; 77.18] | [-1.23; 0.52] | 8.87 |
| **One ES/study (highest)^c^** | 59 | -0.31 | [-0.46; -0.16] | <0.001 | 71.97 | [63.67; 78.37] | [-1.23; 0.6] | 10.22 |
| **Outliers removed^d^** | 65 | -0.25 | [-0.33; -0.18] | <0.001 | 30.46 | [5.2; 48.99] | [-0.56; 0.06] | 12.77 |
| **Influence Analysis^e^** | 71 | -0.25 | [-0.35; -0.16] | <0.001 | 54.75 | [40.84; 65.39] | [-0.78; 0.27] | 12.77 |
| **Only rob > 2^f^** | 69 | -0.31 | [-0.43; -0.18] | <0.001 | 69.10 | [60.48; 75.84] | [-1.13; 0.52] | 10.44 |
| **Three-Level Model^g^** | 72 | -0.33 | [-0.46; -0.19] | <0.001 | 77.70 | - | [-1.2; 0.54] | 9.62 |
| **Three-Level Model (CHE)^h^** | 72 | -0.33 | [-0.46; -0.19] | <0.001 | 77.80 | - | [-1.2; 0.55] | 9.59 |
| *Note:* | | | | | | | | |
| Excluded effect sizes/studies: | | | | | | | | |
| ^a^ combined (arm-level): Brown 2005 ; Bryan 2017 spi; Bryan 2017 spi enhanced; Pachankis 2022 cbt; Peters 2010 | | | | | | | | |
| ^b^ Britton 2020 (dyn vs. cau; BSS); Brown 2005 (cbt vs. ecau; BSS); Brown 2005 (cbt vs. ecau; BSS); Bryan 2017 (spi vs. ecau; BSS); Bryan 2017 (spi vs. ecau; BSS); Bryan 2017 (spi vs. ecau; BSS); Bryan 2017 (spi vs. ecau; BSS); Bryan 2017 (spi vs. ecau; BSS); Ertl 2011 (other vs. wl; MINI); Pachankis 2022 (cbt vs. supp; SIDAS); Patsiokas 1985 (cbt vs. other ctr; BSS); Peters 2010 (cbt vs. wl; BSS); Wei 2013 (mixed vs. cau; BSS) | | | | | | | | |
| ^c^ Britton 2020 (dyn vs. cau; BSS); Brown 2005 (cbt vs. ecau; BSS); Brown 2005 (cbt vs. ecau; BSS); Bryan 2017 (spi vs. ecau; BSS); Bryan 2017 (spi vs. ecau; BSS); Bryan 2017 (spi vs. ecau; BSS); Bryan 2017 (spi vs. ecau; BSS); Bryan 2017 (spi vs. ecau; BSS); Ertl 2011 (img vs. wl; MINI); Pachankis 2022 (cbt vs. cau; SIDAS); Patsiokas 1985 (pst vs. other ctr; BSS); Peters 2010 (cbt vs. wl; BSS); Wei 2013 (cbt vs. cau; BSS) | | | | | | | | |
| ^d^ Britton 2020 (dyn vs. cau; BSS); Fereidouni 2019 (img vs. cau; BSS); LaCroix 2018 (cbt vs. cau; BSS); O'Toole 2019 (other vs. cau; Suicide Status Form); Rahnama 2016 (img vs. cau; BSS); Samaraweera 2007 (cbt vs. cau; BSS); Sedghy 2022 (cbt vs. cau; BSS) | | | | | | | | |
| ^e^ removed as influential cases: Fereidouni 2019 (img vs. cau; BSS) | | | | | | | | |
| ^f^ Brown 2005 (cbt vs. ecau; BSS); Brown 2005 (cbt vs. ecau; BSS); Pistorello 2020 (cams vs. cau; BSS) | | | | | | | | |
| ^g^ Number of clusters/studies: 59; robust variance estimation (RVE) used. | | | | | | | | |
| ^h^ Number of clusters/studies: 59; robust variance estimation (RVE) used. | | | | | | | | |

| **Individual - Attempts** | ***k*** | ***RR*** | **CI** | ***p*** | ***I*^2^** | **CI** | **PI** | **NNT** |
| --- | --- | --- | --- | --- | --- | --- | --- | --- |
| **Overall** | 63 | 0.75 | [0.66; 0.85] | <0.001 | 15.88 | [0; 39.08] | [0.48; 1.18] | - |
| **Combined^a^** | 60 | 0.75 | [0.66; 0.86] | <0.001 | 16.91 | [0; 40.26] | [0.48; 1.19] | - |
| **One ES/study (lowest)^b^** | 56 | 0.75 | [0.65; 0.85] | <0.001 | 18.86 | [0; 42.28] | [0.46; 1.2] | - |
| **One ES/study (highest)^c^** | 56 | 0.77 | [0.67; 0.88] | <0.001 | 19.54 | [0; 42.75] | [0.48; 1.22] | - |
| **Outliers removed^d^** | 62 | 0.73 | [0.64; 0.82] | <0.001 | 6.57 | [0; 31.61] | [0.5; 1.05] | - |
| **Influence Analysis^e^** | 59 | 0.74 | [0.64; 0.85] | <0.001 | 1.26 | [0; 31.6] | [0.51; 1.07] | - |
| **Only rob > 2^f^** | 53 | 0.70 | [0.61; 0.8] | <0.001 | 3.38 | [0; 28.3] | [0.5; 0.98] | - |
| **Three-Level Model^g^** | 63 | 0.75 | [0.65; 0.87] | <0.001 | 22.10 | - | [0.48; 1.19] | - |
| **Three-Level Model (CHE)^h^** | 63 | 0.75 | [0.65; 0.87] | <0.001 | 23.60 | - | [0.47; 1.21] | - |
| *Note:* | | | | | | | | |
| Excluded effect sizes/studies: | | | | | | | | |
| ^a^ combined (arm-level): Amianto 2011 ; Brown 2019 study 2 ; Hetrick 2017 | | | | | | | | |
| ^b^ Amianto 2011 (dyn vs. cau; Clinical Global Impression-Modified); Andreoli 2016 (dyn vs. cau; NA); Britton 2020 (dyn vs. cau; BSS); Brown 2019 study 2 (img vs. supp; BSS); Bryan 2017 (spi vs. ecau; BSS); Hetrick 2017 (cbt vs. cau; SIQ); Wei 2013 (mixed vs. cau; BSS) | | | | | | | | |
| ^c^ Amianto 2011 (dyn vs. cau; Clinical Global Impression-Modified); Andreoli 2016 (dyn vs. cau; NA); Britton 2020 (dyn vs. cau; BSS); Brown 2019 study 2 (img vs. supp; BSS); Bryan 2017 (spi vs. ecau; BSS); Hetrick 2017 (cbt vs. cau; SIQ); Wei 2013 (cbt vs. cau; BSS) | | | | | | | | |
| ^d^ Hatcher 2015 (pst vs. cau; NA) | | | | | | | | |
| ^e^ removed as influential cases: Hatcher 2011 (pst vs. cau; BSS); Hatcher 2015 (pst vs. cau; NA); Naidoo 2014 (other vs. cau; NA); Patel 2017 (other vs. cau; NA) | | | | | | | | |
| ^f^ Brown 2005 (cbt vs. ecau; BSS); Cottraux 2009 (cbt vs. supp; checklist constructed to rate self-harming behaviours (SHBCL) resulting from impulsivity); Doering 2010 (dyn vs. ecau; Cornell Interview for Suicidal and Self-Harming Behavior - Self Report); Gregory 2008 (dyn vs. cau; Lifetime Parasuicide Count); Hatcher 2015 (pst vs. cau; NA); Hvid 2011 (other vs. cau; NA); Ilgen 2023 (cbt vs. other ctr; c-ssrs); Morthorst 2012 (pst vs. cau; Medical records and death register); Tyrer 2003 (cbt vs. cau; PHI); Van der Sande 1997 (other vs. cau; regular visits by the research assistant to all accident and emergency departments of hospitals in the region. Deaths were identified from municipal records and from information obtained from the general practitioner) | | | | | | | | |
| ^g^ Number of clusters/studies: 56; robust variance estimation (RVE) used. | | | | | | | | |
| ^h^ Number of clusters/studies: 56; robust variance estimation (RVE) used. | | | | | | | | |

| **Group - Ideation** | ***k*** | ***g*** | **CI** | ***p*** | ***I*^2^** | **CI** | **PI** | **NNT** |
| --- | --- | --- | --- | --- | --- | --- | --- | --- |
| **Overall** | 27 | -0.46 | [-0.71; -0.21] | <0.001 | 79.43 | [70.7; 85.56] | [-1.59; 0.68] | 6.68 |
| **Combined^a^** | 26 | -0.40 | [-0.62; -0.17] | 0.001 | 76.68 | [66.12; 83.94] | [-1.38; 0.59] | 7.80 |
| **One ES/study (lowest)^b^** | 26 | -0.40 | [-0.63; -0.17] | 0.001 | 76.50 | [65.83; 83.83] | [-1.39; 0.59] | 7.79 |
| **One ES/study (highest)^c^** | 26 | -0.37 | [-0.58; -0.16] | 0.001 | 72.10 | [58.72; 81.14] | [-1.22; 0.48] | 8.38 |
| **Outliers removed^d^** | 23 | -0.27 | [-0.4; -0.15] | <0.001 | 44.90 | [9.96; 66.28] | [-0.7; 0.15] | 11.76 |
| **Influence Analysis^e^** | 26 | -0.37 | [-0.58; -0.16] | 0.001 | 72.10 | [58.72; 81.14] | [-1.22; 0.48] | 8.38 |
| **Only rob > 2^f^** | 26 | -0.47 | [-0.73; -0.21] | 0.001 | 80.20 | [71.71; 86.15] | [-1.64; 0.71] | 6.49 |
| **Three-Level Model^g^** | 27 | -0.41 | [-0.63; -0.18] | 0.001 | 84.70 | - | [-1.45; 0.63] | 7.60 |
| **Three-Level Model (CHE)^h^** | 27 | -0.39 | [-0.6; -0.18] | <0.001 | 82.40 | - | [-1.35; 0.57] | 7.90 |
| *Note:* | | | | | | | | |
| Excluded effect sizes/studies: | | | | | | | | |
| ^a^ combined (arm-level): Nakimuli-Mpungu 2020 | | | | | | | | |
| ^b^ Nakimuli-Mpungu 2020 (cbt vs. other ctr; SAD PERSONS scale) | | | | | | | | |
| ^c^ Nakimuli-Mpungu 2020 (cbt vs. other ctr; SAD PERSONS scale) | | | | | | | | |
| ^d^ Ardashir 2018 (cbt vs. cau; BSS); Nakimuli-Mpungu 2020 (cbt vs. other ctr; SAD PERSONS scale); Nakimuli-Mpungu 2020 (cbt vs. other ctr; SAD PERSONS scale); Rudd 1996 (other vs. cau; M-BSS) | | | | | | | | |
| ^e^ removed as influential cases: Nakimuli-Mpungu 2020 (cbt vs. other ctr; SAD PERSONS scale) | | | | | | | | |
| ^f^ De Jonge-Heesen 2020 (cbt vs. other ctr; VOZZ) | | | | | | | | |
| ^g^ Number of clusters/studies: 26; robust variance estimation (RVE) used. | | | | | | | | |
| ^h^ Number of clusters/studies: 26; robust variance estimation (RVE) used. | | | | | | | | |

| **Group - Attempts** | ***k*** | ***RR*** | **CI** | ***p*** | ***I*^2^** | **CI** | **PI** | **NNT** |
| --- | --- | --- | --- | --- | --- | --- | --- | --- |
| **Overall** | 11 | 0.92 | [0.74; 1.16] | 0.454 | 0 | [0; 60.23] | [0.67; 1.28] | - |
| **Combined^a^** | 10 | 0.92 | [0.72; 1.18] | 0.478 | 0 | [0; 62.37] | [0.66; 1.29] | - |
| **One ES/study (lowest)^b^** | 10 | 0.92 | [0.72; 1.18] | 0.477 | 0 | [0; 62.37] | [0.66; 1.29] | - |
| **One ES/study (highest)^c^** | 10 | 0.92 | [0.72; 1.18] | 0.477 | 0 | [0; 62.37] | [0.66; 1.29] | - |
| **Outliers removed^d^** | 11 | 0.92 | [0.74; 1.16] | 0.454 | 0 | [0; 60.23] | [0.67; 1.28] | - |
| **Influence Analysis^e^** | 11 | 0.92 | [0.74; 1.16] | 0.454 | 0 | [0; 60.23] | [0.67; 1.28] | - |
| **Only rob > 2^f^** | 9 | 0.97 | [0.77; 1.22] | 0.752 | 0 | [0; 64.8] | [0.68; 1.37] | - |
| **Three-Level Model^g^** | 11 | 0.92 | [0.42; 2.02] | 0.663 | 0 | - | [0.66; 1.29] | - |
| **Three-Level Model (CHE)^h^** | 11 | 0.92 | [0.4; 2.15] | 0.673 | 0 | - | [0.65; 1.31] | - |
| *Note:* | | | | | | | | |
| Excluded effect sizes/studies: | | | | | | | | |
| ^a^ combined (arm-level): Nakimuli-Mpungu 2020 | | | | | | | | |
| ^b^ Nakimuli-Mpungu 2020 (cbt vs. other ctr; SAD PERSONS scale) | | | | | | | | |
| ^c^ Nakimuli-Mpungu 2020 (cbt vs. other ctr; SAD PERSONS scale) | | | | | | | | |
| ^d^ no outliers detected | | | | | | | | |
| ^e^ removed as influential cases: none | | | | | | | | |
| ^f^ McMain 2017 (dbt vs. ecau; SASII); Van Wel 2009 (other vs. ecau; bpdsi-iv) | | | | | | | | |
| ^g^ Number of clusters/studies: 10; robust variance estimation (RVE) used. | | | | | | | | |
| ^h^ Number of clusters/studies: 10; robust variance estimation (RVE) used. | | | | | | | | |

| **Family based - Ideation** | ***k*** | ***g*** | **CI** | ***p*** | ***I*^2^** | **CI** | **PI** | **NNT** |
| --- | --- | --- | --- | --- | --- | --- | --- | --- |
| **Overall** | 10 | -0.15 | [-0.38; 0.08] | 0.172 | 43.82 | [0; 73.06] | [-0.5; 0.21] | 22.62 |
| **Combined^a^** | 10 | -0.15 | [-0.38; 0.08] | 0.172 | 43.82 | [0; 73.06] | [-0.5; 0.21] | 22.62 |
| **One ES/study (lowest)** | 10 | -0.15 | [-0.38; 0.08] | 0.172 | 43.82 | [0; 73.06] | [-0.5; 0.21] | 22.62 |
| **One ES/study (highest)** | 10 | -0.15 | [-0.38; 0.08] | 0.172 | 43.82 | [0; 73.06] | [-0.5; 0.21] | 22.62 |
| **Outliers removed^b^** | 10 | -0.15 | [-0.38; 0.08] | 0.172 | 43.82 | [0; 73.06] | [-0.5; 0.21] | 22.62 |
| **Influence Analysis^c^** | 9 | -0.08 | [-0.26; 0.1] | 0.309 | 15.23 | [0; 57.35] | [-0.25; 0.08] | 40.71 |
| **Only rob > 2^d^** | 10 | -0.15 | [-0.38; 0.08] | 0.172 | 43.82 | [0; 73.06] | [-0.5; 0.21] | 22.62 |
| **Three-Level Model^e^** | 10 | -0.15 | [-0.34; 0.04] | 0.106 | 26.60 | - | [-0.5; 0.21] | 22.62 |
| **Three-Level Model (CHE)^f^** | 10 | -0.15 | [-0.34; 0.04] | 0.106 | 26.60 | - | [-0.5; 0.21] | 22.62 |
| *Note:* | | | | | | | | |
| Excluded effect sizes/studies: | | | | | | | | |
| ^a^ combined (arm-level): none | | | | | | | | |
| ^b^ no outliers detected | | | | | | | | |
| ^c^ removed as influential cases: Diamond 2010 (fam vs. ecau; BSS) | | | | | | | | |
| ^d^ no studies removed; rob > 2 applies to all studies | | | | | | | | |
| ^e^ Number of clusters/studies: 10; robust variance estimation (RVE) used. | | | | | | | | |
| ^f^ Number of clusters/studies: 10; robust variance estimation (RVE) used. | | | | | | | | |

| **Family based - Attempts** | ***k*** | ***RR*** | **CI** | ***p*** | ***I*^2^** | **CI** | **PI** | **NNT** |
| --- | --- | --- | --- | --- | --- | --- | --- | --- |
| **Overall** | 10 | 0.81 | [0.46; 1.45] | 0.440 | 37.26 | [0; 70.09] | [0.27; 2.45] | - |
| **Combined^a^** | 10 | 0.81 | [0.46; 1.45] | 0.440 | 37.26 | [0; 70.09] | [0.27; 2.45] | - |
| **One ES/study (lowest)** | 10 | 0.81 | [0.46; 1.45] | 0.440 | 37.26 | [0; 70.09] | [0.27; 2.45] | - |
| **One ES/study (highest)** | 10 | 0.81 | [0.46; 1.45] | 0.440 | 37.26 | [0; 70.09] | [0.27; 2.45] | - |
| **Outliers removed^b^** | 10 | 0.81 | [0.46; 1.45] | 0.440 | 37.26 | [0; 70.09] | [0.27; 2.45] | - |
| **Influence Analysis^c^** | 7 | 0.61 | [0.25; 1.49] | 0.226 | 23.14 | [0; 65.9] | [0.14; 2.71] | - |
| **Only rob > 2^d^** | 10 | 0.81 | [0.46; 1.45] | 0.440 | 37.26 | [0; 70.09] | [0.27; 2.45] | - |
| **Three-Level Model^e^** | 10 | 0.81 | [0.44; 1.5] | 0.431 | 38.40 | - | [0.27; 2.45] | - |
| **Three-Level Model (CHE)^f^** | 10 | 0.81 | [0.44; 1.5] | 0.431 | 38.40 | - | [0.27; 2.45] | - |
| *Note:* | | | | | | | | |
| Excluded effect sizes/studies: | | | | | | | | |
| ^a^ combined (arm-level): none | | | | | | | | |
| ^b^ no outliers detected | | | | | | | | |
| ^c^ removed as influential cases: Cottrell 2018 (fam vs. cau; BSS); Huey 2004 (fam vs. ecau; Youth Risk Behavior Survey); Miklowitz 2020 (fam vs. other ctr; Suicidal Ideation Questionnaire (SIQ) Junior Version; PSR-SI) | | | | | | | | |
| ^d^ no studies removed; rob > 2 applies to all studies | | | | | | | | |
| ^e^ Number of clusters/studies: 10; robust variance estimation (RVE) used. | | | | | | | | |
| ^f^ Number of clusters/studies: 10; robust variance estimation (RVE) used. | | | | | | | | |

| **Individual and group - Ideation** | ***k*** | ***g*** | **CI** | ***p*** | ***I*^2^** | **CI** | **PI** | **NNT** |
| --- | --- | --- | --- | --- | --- | --- | --- | --- |
| **Overall** | 7 | -0.33 | [-0.71; 0.04] | 0.074 | 56.42 | [0; 81.24] | [-1.08; 0.42] | 9.54 |
| **Combined^a^** | 7 | -0.33 | [-0.71; 0.04] | 0.074 | 56.42 | [0; 81.24] | [-1.08; 0.42] | 9.54 |
| **One ES/study (lowest)** | 7 | -0.33 | [-0.71; 0.04] | 0.074 | 56.42 | [0; 81.24] | [-1.08; 0.42] | 9.54 |
| **One ES/study (highest)** | 7 | -0.33 | [-0.71; 0.04] | 0.074 | 56.42 | [0; 81.24] | [-1.08; 0.42] | 9.54 |
| **Outliers removed^b^** | 7 | -0.33 | [-0.71; 0.04] | 0.074 | 56.42 | [0; 81.24] | [-1.08; 0.42] | 9.54 |
| **Influence Analysis^c^** | 7 | -0.33 | [-0.71; 0.04] | 0.074 | 56.42 | [0; 81.24] | [-1.08; 0.42] | 9.54 |
| **Only rob > 2^d^** | 7 | -0.33 | [-0.71; 0.04] | 0.074 | 56.42 | [0; 81.24] | [-1.08; 0.42] | 9.54 |
| **Three-Level Model^e^** | 7 | -0.33 | [-0.69; 0.03] | 0.063 | 57.60 | - | [-1.08; 0.42] | 9.54 |
| **Three-Level Model (CHE)^f^** | 7 | -0.33 | [-0.69; 0.03] | 0.063 | 57.60 | - | [-1.08; 0.42] | 9.54 |
| *Note:* | | | | | | | | |
| Excluded effect sizes/studies: | | | | | | | | |
| ^a^ combined (arm-level): none | | | | | | | | |
| ^b^ no outliers detected | | | | | | | | |
| ^c^ removed as influential cases: none | | | | | | | | |
| ^d^ no studies removed; rob > 2 applies to all studies | | | | | | | | |
| ^e^ Number of clusters/studies: 7; robust variance estimation (RVE) used. | | | | | | | | |
| ^f^ Number of clusters/studies: 7; robust variance estimation (RVE) used. | | | | | | | | |

| **Individual and Group - Attempts** | ***k*** | ***RR*** | **CI** | ***p*** | ***I*^2^** | **CI** | **PI** | **NNT** |
| --- | --- | --- | --- | --- | --- | --- | --- | --- |
| **Overall** | 7 | 0.42 | [0.25; 0.71] | 0.007 | 27.21 | [0; 68.49] | [0.27; 0.66] | - |
| **Combined^a^** | 7 | 0.42 | [0.25; 0.71] | 0.007 | 27.21 | [0; 68.49] | [0.27; 0.66] | - |
| **One ES/study (lowest)** | 7 | 0.42 | [0.25; 0.71] | 0.007 | 27.21 | [0; 68.49] | [0.27; 0.66] | - |
| **One ES/study (highest)** | 7 | 0.42 | [0.25; 0.71] | 0.007 | 27.21 | [0; 68.49] | [0.27; 0.66] | - |
| **Outliers removed^b^** | 7 | 0.42 | [0.25; 0.71] | 0.007 | 27.21 | [0; 68.49] | [0.27; 0.66] | - |
| **Influence Analysis^c^** | 5 | 0.31 | [0.09; 1.05] | 0.056 | 47.59 | [0; 80.8] | [0.03; 2.96] | - |
| **Only rob > 2^d^** | 4 | 0.51 | [0.35; 0.73] | 0.010 | 0.00 | [0; 84.69] | [0.27; 0.95] | - |
| **Three-Level Model^e^** | 7 | 0.42 | [0.27; 0.67] | 0.010 | 0.00 | - | [0.3; 0.6] | - |
| **Three-Level Model (CHE)^f^** | 7 | 0.42 | [0.27; 0.67] | 0.010 | 0.00 | - | [0.3; 0.6] | - |
| *Note:* | | | | | | | | |
| Excluded effect sizes/studies: | | | | | | | | |
| ^a^ combined (arm-level): none | | | | | | | | |
| ^b^ no outliers detected | | | | | | | | |
| ^c^ removed as influential cases: Interian 2021 (mbct vs. ecau; BSS); Linehan 2006 (dbt vs. ecau; SBQ) | | | | | | | | |
| ^d^ Bateman 1999 (dyn vs. cau; SSI); Bateman 2009 (mbt vs. ecau; NA); Verheul 2003 (dbt vs. cau; bpdsi-iv) | | | | | | | | |
| ^e^ Number of clusters/studies: 7; robust variance estimation (RVE) used. | | | | | | | | |
| ^f^ Number of clusters/studies: 7; robust variance estimation (RVE) used. | | | | | | | | |

| **Individual and Family based - Ideation** | ***k*** | ***g*** | **CI** | ***p*** | ***I*^2^** | **CI** | **PI** | **NNT** |
| --- | --- | --- | --- | --- | --- | --- | --- | --- |
| **Overall** | 12 | -0.39 | [-0.83; 0.04] | 0.074 | 74.97 | [55.9; 85.8] | [-1.53; 0.74] | 7.85 |
| **Combined^a^** | 9 | -0.43 | [-1.13; 0.28] | 0.202 | 74.16 | [49.81; 86.69] | [-2.32; 1.47] | 7.19 |
| **One ES/study (lowest)^b^** | 8 | -0.47 | [-1.3; 0.36] | 0.223 | 79.35 | [59.72; 89.41] | [-2.65; 1.71] | 6.45 |
| **One ES/study (highest)^c^** | 8 | -0.40 | [-1.23; 0.43] | 0.295 | 76.75 | [53.72; 88.32] | [-2.56; 1.77] | 7.80 |
| **Outliers removed^d^** | 11 | -0.33 | [-0.48; -0.18] | <0.001 | 52.54 | [5.86; 76.08] | [-0.68; 0.02] | 9.56 |
| **Influence Analysis^e^** | 12 | -0.39 | [-0.83; 0.04] | 0.074 | 74.97 | [55.9; 85.8] | [-1.53; 0.74] | 7.85 |
| **Only rob > 2^f^** | 12 | -0.39 | [-0.83; 0.04] | 0.074 | 74.97 | [55.9; 85.8] | [-1.53; 0.74] | 7.85 |
| **Three-Level Model^g^** | 12 | -0.43 | [-1.2; 0.34] | 0.228 | 97.80 | - | [-2.41; 1.55] | 7.13 |
| **Three-Level Model (CHE)^h^** | 12 | -0.43 | [-1.21; 0.35] | 0.231 | 97.80 | - | [-2.42; 1.56] | 7.09 |
| *Note:* | | | | | | | | |
| Excluded effect sizes/studies: | | | | | | | | |
| ^a^ combined (arm-level): Husain 2023 | | | | | | | | |
| ^b^ Brent 1997 (cbt vs. supp; K-SADS-P/E); Husain 2023 (pst vs. cau; bssi); Husain 2023 (pst vs. cau; bssi); Husain 2023 (pst vs. cau; bssi) | | | | | | | | |
| ^c^ Brent 1997 (fam vs. supp; K-SADS-P/E); Husain 2023 (pst vs. cau; bssi); Husain 2023 (pst vs. cau; bssi); Husain 2023 (pst vs. cau; bssi) | | | | | | | | |
| ^d^ Alavi 2013 (cbt vs. wl; BSS) | | | | | | | | |
| ^e^ removed as influential cases: none | | | | | | | | |
| ^f^ no studies removed; rob > 2 applies to all studies | | | | | | | | |
| ^g^ Number of clusters/studies: 8; robust variance estimation (RVE) used. | | | | | | | | |
| ^h^ Number of clusters/studies: 8; robust variance estimation (RVE) used. | | | | | | | | |

| **Individual and family based - Attempts** | ***k*** | ***RR*** | **CI** | ***p*** | ***I*^2^** | **CI** | **PI** | **NNT** |
| --- | --- | --- | --- | --- | --- | --- | --- | --- |
| **Overall** | 8 | 0.59 | [0.42; 0.81] | 0.006 | 0 | [0; 67.58] | [0.35; 0.97] | - |
| **Combined^a^** | 8 | 0.59 | [0.42; 0.81] | 0.006 | 0 | [0; 67.58] | [0.35; 0.97] | - |
| **One ES/study (lowest)** | 8 | 0.59 | [0.42; 0.81] | 0.006 | 0 | [0; 67.58] | [0.35; 0.97] | - |
| **One ES/study (highest)** | 8 | 0.59 | [0.42; 0.81] | 0.006 | 0 | [0; 67.58] | [0.35; 0.97] | - |
| **Outliers removed^b^** | 8 | 0.59 | [0.42; 0.81] | 0.006 | 0 | [0; 67.58] | [0.35; 0.97] | - |
| **Influence Analysis^c^** | 7 | 0.50 | [0.33; 0.77] | 0.007 | 0 | [0; 70.81] | [0.25; 1] | - |
| **Only rob > 2^d^** | 7 | 0.50 | [0.33; 0.77] | 0.007 | 0 | [0; 70.81] | [0.25; 1] | - |
| **Three-Level Model^e^** | 8 | 0.59 | [0.36; 0.96] | 0.041 | 0 | - | [0.42; 0.82] | - |
| **Three-Level Model (CHE)^f^** | 8 | 0.59 | [0.36; 0.96] | 0.041 | 0 | - | [0.42; 0.82] | - |
| *Note:* | | | | | | | | |
| Excluded effect sizes/studies: | | | | | | | | |
| ^a^ combined (arm-level): none | | | | | | | | |
| ^b^ no outliers detected | | | | | | | | |
| ^c^ removed as influential cases: Goldstein 2023 (dbt vs. ecau; c-ssrs) | | | | | | | | |
| ^d^ Goldstein 2023 (dbt vs. ecau; c-ssrs) | | | | | | | | |
| ^e^ Number of clusters/studies: 8; robust variance estimation (RVE) used. | | | | | | | | |
| ^f^ Number of clusters/studies: 8; robust variance estimation (RVE) used. | | | | | | | | |

## Appendix B: Reference list of included studies

1. Alavi, A., Sharifi, B., Ghanizadeh, A., & Dehbozorgi, G. (2013). Effectiveness of cognitive-behavioral therapy in decreasing suicidal ideation and hopelessness of the adolescents with previous suicidal attempts. Iranian journal of pediatrics, 23(4), 467â€“472.
2. Amianto, F., Ferrero, A., PierÃ², A., Cairo, E., Rocca, G., Simonelli, B., Fassina, S., Abbate-Daga, G., & Fassino, S. (2011). Supervised team management, with or without structured psychotherapy, in heavy users of a mental health service with borderline personality disorder: a two-year follow- up preliminary randomized study. BMC psychiatry, 11, 181. https://doi.org/10.1186/1471-244X-11-181
3. Andreoli, A., Burnand, Y., Cochennec, M. F., Ohlendorf, P., Frambati, L., Gaudry-Maire, D., Di Clemente, T., Hourton, G., Lorillard, S., Canuto, A., & Frances, A. (2016). Disappointed Love and Suicide: A Randomized Controlled Trial of "Abandonment Psychotherapy" Among Borderline Patients. Journal of personality disorders, 30(2), 271â€“287. https://doi.org/10.1521/pedi_2015_29_196
4. Afrasiabifar, Ardashir & Hosseini, Nazafarin & Haghgoo, Amin. (2018). The Effects of Group Cognitive Behavior Therapy (GCBT) on Suicidal Thoughts in Patients with Major Depression. World Family Medicine Journal/Middle East Journal of Family Medicine. 16. 228-235. 10.5742/MEWFM.2018.93293.
5. Arnevik, E., Wilberg, T., Urnes, O., Johansen, M., Monsen, J. T., & Karterud,S. (2009). Psychotherapy for personality disorders: short-term day hospital psychotherapy versus outpatient individual therapy - a randomized controlled study. European psychiatry : the journal of the Association of European Psychiatrists, 24(2), 71â€“78. https://doi.org/10.1016/j.eurpsy.2008.09.004
6. Arvilommi, P., Valkonen, J., LIndholm, L. H., Gaily-Luoma, S., Suominen, K., Ruishalme, O. M., ... & IsometsÃ¤, E. (2022). A randomized clinical trial of Attempted Suicide Short Intervention Program versus crisis counseling in preventing repeat suicide attempts: a two-year follow-up study. Psychotherapy and psychosomatics, 91(3), 190-199.
7. Asarnow, J. R., Baraff, L. J., Berk, M., Grob, C. S., Devich-Navarro, M., Suddath, R., Piacentini, J. C., Rotheram-Borus, M. J., Cohen, D., & Tang, L. (2011). An emergency department intervention for linking pediatric suicidal patients to follow-up mental health treatment. Psychiatric services (Washington, D.C.), 62(11), 1303â€“1309. https://doi.org/10.1176/ps.62.11.pss6211_1303
8. Asarnow, J. R., Hughes, J. L., Babeva, K. N., & Sugar, C. A. (2017). Cognitive-Behavioral Family Treatment for Suicide Attempt Prevention: A Randomized Controlled Trial. Journal of the American Academy of Child and Adolescent Psychiatry, 56(6), 506â€“514. https://doi.org/10.1016/j.jaac.2017.03.015
9. Asarnow, J. R., Tompson, M. C., Klomhaus, A. M., Babeva, K., Langer, D. A., & Sugar, C. A. (2020). Randomized controlled trial of familyâ€focused treatment for child depression compared to individual psychotherapy: oneâ€year outcomes.Â Journal of child psychology and psychiatry,Â 61(6), 662-671.
10. Ayar, D., & Sabanciogullari, S. (2021). The effect of a solutionâ€oriented approach in depressive patients on social functioning levels and suicide probability.Â Perspectives in psychiatric care,Â 57(1), 235-245.
11. Bannan, Noreen, (2010), Group-based problem-solving therapy in self- poisoning females: A pilot study. Counselling and Psychotherapy Research, 10 doi: 10.1080/14733140903337292.
12. Barnes, S. M., Borges, L. M., Smith, G. P., Walser, R. D., Forster, J. E., & Bahraini, N. H. (2021). Acceptance and commitment therapy to promote recovery from suicidal crises: A randomized controlled acceptability and feasibility trial of ACT for life.Â Journal of Contextual Behavioral Science,Â 20, 35-45.
13. Barnhofer, T., Crane, C., Hargus, E., Amarasinghe, M., Winder, R., & Williams, J. M. (2009). Mindfulness-based cognitive therapy as a treatment for chronic depression: A preliminary study. Behaviour research and therapy, 47(5), 366â€“373. https://doi.org/10.1016/j.brat.2009.01.019
14. Bateman, A., & Fonagy, P. (1999). Effectiveness of partial hospitalization in the treatment of borderline personality disorder: a randomized controlled trial. The American journal of psychiatry, 156(10), 1563â€“1569. https://doi.org/10.1176/ajp.156.10.1563
15. Bateman, A., & Fonagy, P. (2009). Randomized controlled trial of outpatient mentalization-based treatment versus structured clinical management for borderline personality disorder. The American journal of psychiatry, 166(12), 1355â€“1364. https://doi.org/10.1176/appi.ajp.2009.09040539
16. Blomdahl, C., GuregÃ¥rd, S., Rusner, M., & Wijk, H. (2018). A manual-based phenomenological art therapy for individuals diagnosed with moderate to severe depression (PATd): A randomized controlled study. Psychiatric rehabilitation journal, 41(3), 169â€“182. https://doi.org/10.1037/prj0000300
17. Brenner, L. A., Forster, J. E., Hoffberg, A. S., Matarazzo, B. B., Hostetter, T. A., Signoracci, G., & Simpson, G. K. (2018). Window to Hope: A Randomized Controlled Trial of a Psychological Intervention for the Treatment of Hopelessness Among Veterans With Moderate to Severe Traumatic Brain Injury. The Journal of head trauma rehabilitation, 33(2), E64â€“E73. https://doi.org/10.1097/HTR.0000000000000351
18. Brent, D. A., Holder, D., Kolko, D., Birmaher, B., Baugher, M., Roth, C., Iyengar, S., & Johnson, B. A. (1997). A clinical psychotherapy trial for adolescent depression comparing cognitive, family, and supportive therapy. Archives of general psychiatry, 54(9), 877â€“885. https://doi.org/10.1001/archpsyc.1997.01830210125017
19. Britton, P. C., Conner, K. R., Chapman, B. P., & Maisto, S. A. (2020). Motivational interviewing to address suicidal ideation: A randomized controlled trial in veterans.Â Suicide and Lifeâ€Threatening Behavior,Â 50(1), 233-248.
20. Brown, G. K., Ten Have, T., Henriques, G. R., Xie, S. X., Hollander, J. E., & Beck, A. T. (2005). Cognitive therapy for the prevention of suicide attempts: a randomized controlled trial. JAMA, 294(5), 563â€“570. https://doi.org/10.1001/jama.294.5.563
21. Brown, L. A., McLean, C. P., Zang, Y., Zandberg, L., Mintz, J., Yarvis, J. S., Litz, B. T., Peterson, A. L., Bryan, C. J., Fina, B., Petersen, J., Dondanville, K. A., Roache, J. D., Young-McCaughan, S., Foa, E. B., & STRONG STAR consortium (2019). Does prolonged exposure increase suicide risk? Results from an active duty military sample. Behaviour research and therapy, 118, 87â€“93. https://doi.org/10.1016/j.brat.2019.04.003
22. Brown, L. A., McLean, C. P., Zang, Y., Zandberg, L., Mintz, J., Yarvis, J. S., Litz, B. T., Peterson, A. L., Bryan, C. J., Fina, B., Petersen, J., Dondanville, K. A., Roache, J. D., Young-McCaughan, S., Foa, E. B., & STRONG STAR consortium (2019). Does prolonged exposure increase suicide risk? Results from an active duty military sample. Behaviour research and therapy, 118, 87â€“93. https://doi.org/10.1016/j.brat.2019.04.003
23. Bryan, C. J., Mintz, J., Clemans, T. A., Leeson, B., Burch, T. S., Williams, S. R., Maney, E., & Rudd, M. D. (2017). Effect of crisis response planning vs. contracts for safety on suicide risk in U.S. Army Soldiers: A randomized clinical trial. Journal of affective disorders, 212, 64â€“72. https://doi.org/10.1016/j.jad.2017.01.028
24. Bryant, R., Dawson, K., Azevedo, S., Yadav, S., Tran, J., Choi-Christou, J., ... & Keyan, D. (2023). Positive affect training to reduce mental health problems during the COVID-19 pandemic: a proof-of-concept randomised clinical trial.Â BMJ Ment Health,Â 26(1).
25. Bryant, R., Dawson, K., Azevedo, S., Yadav, S., Tran, J., Choi-Christou, J., ... & Keyan, D. (2023). Positive affect training to reduce mental health problems during the COVID-19 pandemic: a proof-of-concept randomised clinical trial.Â BMJ Ment Health,Â 26(1).
26. Celano, C. M., Beale, E. E., Mastromauro, C. A., Stewart, J. G., Millstein, R. A., Auerbach, R. P., Bedoya, C. A., & Huffman, J. C. (2017). Psychological interventions to reduce suicidality in high-risk patients with major depression: a randomized controlled trial. Psychological medicine, 47(5), 810â€“821. https://doi.org/10.1017/S0033291716002798
27. Chan, N. Y., Lam, S. P., Zhang, J., Chan, J. W. Y., Yu, M. M. W., Suh, S., ... & Li, S. X. (2022). Efficacy of email-delivered versus face-to-face group cognitive behavioral therapy for insomnia in youths: a randomized controlled trial.Â Journal of Adolescent Health,Â 70(5), 763-773.
28. Chanen, A. M., Betts, J. K., Jackson, H., Cotton, S. M., Gleeson, J., Davey, C. G., ... & McCutcheon, L. (2022). Effect of 3 forms of early intervention for young people with borderline personality disorder: The MOBY randomized clinical trial. JAMA psychiatry, 79(2), 109-119.
29. Chen, Y. L., Pan, A. W., Hsiung, P. C., Chung, L., Lai, J. S., Shur-Fen Gau, S., & Chen, T. J. (2015). Life Adaptation Skills Training (LAST) for persons with depression: A randomized controlled study. Journal of affective disorders, 185, 108â€“114. https://doi.org/10.1016/j.jad.2015.06.022
30. Christopher, M., Bowen, S., Witkiewitz, K., Grupe, D., Goerling, R., Hunsinger, M., ... & Rosenbaum, N. (2024). A multisite feasibility randomized clinical trial of mindfulness-based resilience training for aggression, stress, and health in law enforcement officers.Â BMC complementary medicine and therapies,Â 24(1), 142.
31. Comtois, K. A., Jobes, D. A., S O'Connor, S., Atkins, D. C., Janis, K., E Chessen, C., Landes, S. J., Holen, A., & Yuodelis-Flores, C. (2011). Collaborative assessment and management of suicidality (CAMS): feasibility trial for next-day appointment services. Depression and anxiety, 28(11), 963â€“ 972. https://doi.org/10.1002/da.20895
32. Conner, K. R., Kearns, J. C., Esposito, E. C., Pizzarello, E., Wiegand, T. J., Britton, P. C., ... & Goldston, D. B. (2021). Pilot RCT of the Attempted Suicide Short Intervention Program (ASSIP) adapted for rapid delivery during hospitalization to adult suicide attempt patients with substance use problems.Â General hospital psychiatry,Â 72, 66-72.
33. Cottraux, J., Note, I. D., Boutitie, F., Milliery, M., Genouihlac, V., Yao, S. N., Note, B., Mollard, E., Bonasse, F., Gaillard, S., Djamoussian, D., Guillard, C., Culem, A., & Gueyffier, F. (2009). Cognitive therapy versus Rogerian supportive therapy in borderline personality disorder. Two-year follow-up of a controlled pilot study. Psychotherapy and psychosomatics, 78(5), 307â€“316. https://doi.org/10.1159/000229769
34. Cottrell, D. J., Wright-Hughes, A., Collinson, M., Boston, P., Eisler, I., Fortune, S., Graham, E. H., Green, J., House, A. O., Kerfoot, M., Owens, D. W., Saloniki, E. C., Simic, M., Lambert, F., Rothwell, J., Tubeuf, S., & Farrin,J. (2018). Effectiveness of systemic family therapy versus treatment as usual for young people after self-harm: a pragmatic, phase 3, multicentre, randomised controlled trial. The lancet. Psychiatry, 5(3), 203â€“216. https://doi.org/10.1016/S2215-0366(18)30058-0
35. Crawford, M. J., Thana, L., Parker, J., Turner, O., Xing, K. P., McMurran, M., Moran, P., Weaver, T., Barrett, B., Claringbold, A., Bassett, P., & Sanatinia,R. (2018). Psychological Support for Personality (PSP) versus treatment as usual: study protocol for a feasibility randomized controlled trial of a low intensity intervention for people with personality disorder. Trials, 19(1), 547. https://doi.org/10.1186/s13063-018-2920-0
36. Daneshvar, S., Shafiei, M., & Basharpoor, S. (2022). Compassion-focused therapy: Proof of concept trial on suicidal ideation and cognitive distortions in female survivors of intimate partner violence with PTSD. Journal of interpersonal violence, 37(11-12), NP9613-NP9634.
37. Davidson, K., Norrie, J., Tyrer, P., Gumley, A., Tata, P., Murray, H., & Palmer, S. (2006). The effectiveness of cognitive behavior therapy for borderline personality disorder: results from the borderline personality disorder study of cognitive therapy (BOSCOT) trial. Journal of personality disorders, 20(5), 450â€“465. https://doi.org/10.1521/pedi.2006.20.5.450
38. Davidson, K. M., Brown, T. M., James, V., Kirk, J., & Richardson, J. (2014). Manual-assisted cognitive therapy for self-harm in personality disorder and substance misuse: a feasibility trial. Psychiatric bulletin (2014), 38(3), 108â€“ 111. https://doi.org/10.1192/pb.bp.113.043109
39. de Jonge-Heesen, K. W., Rasing, S. P., Vermulst, A. A., Scholte, R. H., Van Ettekoven, K. M., Engels, R. C., & Creemers, D. H. (2021). Secondary outcomes of implemented depression prevention in adolescents: a randomized controlled trial.Â Frontiers in Psychiatry,Â 12, 643632.
40. de Groot, M., de Keijser, J., Neeleman, J., Kerkhof, A., Nolen, W., & Burger,H. (2007). Cognitive behaviour therapy to prevent complicated grief among relatives and spouses bereaved by suicide: cluster randomised controlled trial. BMJ (Clinical research ed.), 334(7601), 994. https://doi.org/10.1136/bmj.39161.457431.55
41. De Jaegere, E., Stas, P., Van Heeringen, K., Dumon, E., van Landschoot, R., & Portzky, G. (2023). Futureâ€Oriented Group Training for suicidal individuals: A randomized controlled trial.Â Suicide and Lifeâ€Threatening Behavior,Â 53(2), 270-281.
42. Diamond, G. S., Wintersteen, M. B., Brown, G. K., Diamond, G. M., Gallop, R., Shelef, K., & Levy, S. (2010). Attachment-based family therapy for adolescents with suicidal ideation: a randomized controlled trial. Journal of the American Academy of Child and Adolescent Psychiatry, 49(2), 122â€“131. https://doi.org/10.1097/00004583-201002000-00006
43. Diamond, G. S., Kobak, R. R., Krauthamer Ewing, E. S., Levy, S. A., Herres,J. L., Russon, J. M., & Gallop, R. J. (2019). A Randomized Controlled Trial: Attachment-Based Family and Nondirective Supportive Treatments for Youth Who Are Suicidal. Journal of the American Academy of Child and Adolescent Psychiatry, 58(7), 721â€“731. https://doi.org/10.1016/j.jaac.2018.10.006
44. Doering, S., HÃ¶rz, S., Rentrop, M., Fischer-Kern, M., Schuster, P., Benecke, C., Buchheim, A., Martius, P., & Buchheim, P. (2010). Transference-focused psychotherapy v. treatment by community psychotherapists for borderline personality disorder: randomised controlled trial. The British journal of psychiatry : the journal of mental science, 196(5), 389â€“395. https://doi.org/10.1192/bjp.bp.109.070177
45. Donaldson, D., Spirito, A., & Esposito-Smythers, C. (2005). Treatment for adolescents following a suicide attempt: results of a pilot trial. Journal of the American Academy of Child and Adolescent Psychiatry, 44(2), 113â€“120. https://doi.org/10.1097/00004583-200502000-00003
46. Ducasse, D., Jaussent, I., Arpon-Brand, V., Vienot, M., Laglaoui, C., BÃ©ziat, S., Calati, R., CarriÃ¨re, I., Guillaume, S., Courtet, P., & OliÃ©, E. (2018). Acceptance and Commitment Therapy for the Management of Suicidal Patients: A Randomized Controlled Trial. Psychotherapy and psychosomatics, 87(4), 211â€“222. https://doi.org/10.1159/000488715
47. Ducasse, D., Dassa, D., Courtet, P., Brand-Arpon, V., Walter, A., Guillaume, S., Jaussent, I., & OliÃ©, E. (2019). Gratitude diary for the management of suicidal inpatients: A randomized controlled trial. Depression and anxiety, 36(5), 400â€“411. https://doi.org/10.1002/da.22877
48. Ertl, V., Pfeiffer, A., Schauer, E., Elbert, T., & Neuner, F. (2011). Community-implemented trauma therapy for former child soldiers in Northern Uganda: a randomized controlled trial. JAMA, 306(5), 503â€“512. https://doi.org/10.1001/jama.2011.1060
49. Eskin, M., Ertekin, K. & Demir, H. Efficacy of a Problem-Solving Therapy for Depression and Suicide Potential in Adolescents and Young Adults. Cogn Ther Res 32, 227â€“245 (2008). https://doi.org/10.1007/s10608-007-9172-8
50. Esposito-Smythers, C., Spirito, A., Kahler, C. W., Hunt, J., & Monti, P. (2011). Treatment of co-occurring substance abuse and suicidality among adolescents: a randomized trial. Journal of consulting and clinical psychology, 79(6), 728â€“739. https://doi.org/10.1037/a0026074
51. Esposito-Smythers, C., Hadley, W., Curby, T. W., & Brown, L. K. (2017). Randomized pilot trial of a cognitive-behavioral alcohol, self-harm, and HIV prevention program for teens in mental health treatment. Behaviour research and therapy, 89, 49â€“56. https://doi.org/10.1016/j.brat.2016.11.005
52. Esposito-Smythers, C., Wolff, J. C., Liu, R. T., Hunt, J. I., Adams, L., Kim, K., Frazier, E. A., Yen, S., Dickstein, D. P., & Spirito, A. (2019). Family-focused cognitive behavioral treatment for depressed adolescents in suicidal crisis with co-occurring risk factors: a randomized trial. Journal of child psychology and psychiatry, and allied disciplines, 60(10), 1133â€“1141. https://doi.org/10.1111/jcpp.13095
53. Evans, K., Tyrer, P., Catalan, J., Schmidt, U., Davidson, K., Dent, J., Tata, P., Thornton, S., Barber, J., & Thompson, S. (1999). Manual-assisted cognitive- behaviour therapy (MACT): a randomized controlled trial of a brief intervention with bibliotherapy in the treatment of recurrent deliberate self- harm. Psychological medicine, 29(1), 19â€“25. https://doi.org/10.1017/s003329179800765x
54. Eylem, O., van Straten, A., Bhui, K., & Kerkhof, A. J. (2015). Protocol: Reducing suicidal ideation among Turkish migrants in the Netherlands and in the UK: effectiveness of an online intervention. International review of psychiatry (Abingdon, England), 27(1), 72â€“81. https://doi.org/10.3109/09540261.2014.996121
55. FÃ¤rdig, R., Lewander, T., Melin, L., Folke, F., & Fredriksson, A. (2011). A randomized controlled trial of the illness management and recovery program for persons with schizophrenia. Psychiatric services (Washington, D.C.), 62(6), 606â€“612. https://doi.org/10.1176/ps.62.6.pss6206_0606
56. Fereidouni, Z., Behnammoghadam, M., Jahanfar, A., & Dehghan, A. (2019). The Effect of Eye Movement Desensitization and Reprocessing (EMDR) on the severity of suicidal thoughts in patients with major depressive disorder: a randomized controlled trial.Â Neuropsychiatric disease and treatment, 2459-2466.
57. Ghahramanlou-Holloway, M., LaCroix, J. M., Perera, K. U., Neely, L., Grammer, G., Weaver, J., ... & Lee-Tauler, S. Y. (2020). Inpatient psychiatric care following a suicide-related hospitalization: A pilot trial of Post-Admission Cognitive Therapy in a military medical center.Â General Hospital Psychiatry,Â 63, 46-53.
58. Goldstein, T. R., Fersch-Podrat, R. K., Rivera, M., Axelson, D. A., Merranko, J., Yu, H., Brent, D. A., & Birmaher, B. (2015). Dialectical behavior therapy for adolescents with bipolar disorder: results from a pilot randomized trial. Journal of child and adolescent psychopharmacology, 25(2), 140â€“149. https://doi.org/10.1089/cap.2013.0145
59. Goldstein, T. R., Merranko, J., Rode, N., Sylvester, R., Hotkowski, N., Fersch-Podrat, R., ... & Birmaher, B. (2024). Dialectical behavior therapy for adolescents with bipolar disorder: A Randomized Clinical Trial.Â JAMA psychiatry,Â 81(1), 15-24.
60. Goldston, D. B., Curry, J. F., Wells, K. C., Kaminer, Y., Daniel, S. S., Esposito-Smythers, C., ... & Roley-Roberts, M. (2021). Feasibility of an integrated treatment approach for youth with depression, suicide attempts, and substance use problems. Evidence-based practice in child and adolescent mental health, 6(2), 155-172.
61. Goodman, M., Banthin, D., Blair, N. J., Mascitelli, K. A., Wilsnack, J., Chen, J., Messenger, J. W., Perez-Rodriguez, M. M., Triebwasser, J., Koenigsberg,H. W., Goetz, R. R., Hazlett, E. A., & New, A. S. (2016). A Randomized Trial of Dialectical Behavior Therapy in High-Risk Suicidal Veterans. The Journal of clinical psychiatry, 77(12), e1591â€“e1600. https://doi.org/10.4088/JCP.15m10235
62. Goodman, M., Sullivan, S. R., Spears, A. P., Crasta, D., Mitchell, E. L., Stanley, B., ... & Glynn, S. (2022). A pilot randomized control trial of a dyadic safety planning intervention: Safe actions for families to encourage recovery.Â Couple and Family Psychology: Research and Practice,Â 11(1), 42.
63. Goodyer, I. M., Dubicka, B., Wilkinson, P., Kelvin, R., Roberts, C., Byford, S., Breen, S., Ford, C., Barrett, B., Leech, A., Rothwell, J., White, L., & Harrington, R. (2008). A randomised controlled trial of cognitive behaviour therapy in adolescents with major depression treated by selective serotonin reuptake inhibitors. The ADAPT trial. Health technology assessment (Winchester, England), 12(14), iiiâ€“60. https://doi.org/10.3310/hta12140
64. Green, J. M., Wood, A. J., Kerfoot, M. J., Trainor, G., Roberts, C., Rothwell, J., Woodham, A., Ayodeji, E., Barrett, B., Byford, S., & Harrington, R. (2011). Group therapy for adolescents with repeated self harm: randomised controlled trial with economic evaluation. BMJ (Clinical research ed.), 342, d682. https://doi.org/10.1136/bmj.d682
65. Gregory, R. J., Chlebowski, S., Kang, D., Remen, A. L., Soderberg, M. G., Stepkovitch, J., & Virk, S. (2008). A controlled trial of psychodynamic psychotherapy for co-occurring borderline personality disorder and alcohol use disorder. Psychotherapy (Chicago, Ill.), 45(1), 28â€“41. https://doi.org/10.1037/0033-3204.45.1.28
66. Grupp-Phelan, J., Stevens, J., Boyd, S., Cohen, D. M., Ammerman, R. T., Liddy-Hicks, S., ... & Bridge, J. A. (2019). Effect of a motivational interviewingâ€“based intervention on initiation of mental health treatment and mental health after an emergency department visit among suicidal adolescents: a randomized clinical trial.Â JAMA network open,Â 2(12), e1917941-e1917941.
67. Guthrie, E., Kapur, N., Mackway-Jones, K., Chew-Graham, C., Moorey, J., Mendel, E., Marino-Francis, F., Sanderson, S., Turpin, C., Boddy, G., & Tomenson, B. (2001). Randomised controlled trial of brief psychological intervention after deliberate self poisoning. BMJ (Clinical research ed.), 323(7305), 135â€“138. https://doi.org/10.1136/bmj.323.7305.135
68. Gutierrez, P. M., Johnson, L., Podlogar, M. C., Hagman, S., Muehler, T. A., Hanson, J., ... & Oâ€™Connor, S. (2022). Pilot study of the Collaborative Assessment and Management of Suicidalityâ€”Group.Â Suicide and Lifeâ€Threatening Behavior,Â 52(2), 244-255.
69. Gysin-Maillart, A., Schwab, S., Soravia, L., Megert, M., & Michel, K. (2016). A Novel Brief Therapy for Patients Who Attempt Suicide: A 24-months Follow-Up Randomized Controlled Study of the Attempted Suicide Short Intervention Program (ASSIP). PLoS medicine, 13(3), e1001968. https://doi.org/10.1371/journal.pmed.1001968
70. Haddock, G., Pratt, D., Gooding, P. A., Peters, S., Emsley, R., Evans, E., Kelly, J., Huggett, C., Munro, A., Harris, K., Davies, L., & Awenat, Y. (2019). Feasibility and acceptability of suicide prevention therapy on acute psychiatric wards: randomised controlled trial. BJPsych open, 5(1), e14. https://doi.org/10.1192/bjo.2018.85
71. Hahm, H. C., Zhou, L., Lee, C., Maru, M., Petersen, J. M., & Kolaczyk, E. D. (2019). Feasibility, preliminary efficacy, and safety of a randomized clinical trial for Asian Women's Action for Resilience and Empowerment (AWARE) intervention. The American journal of orthopsychiatry, 89(4), 462â€“474. https://doi.org/10.1037/ort0000383
72. Harrington, R., Kerfoot, M., Dyer, E., McNiven, F., Gill, J., Harrington, V., Woodham, A., & Byford, S. (1998). Randomized trial of a home-based family intervention for children who have deliberately poisoned themselves. Journal of the American Academy of Child and Adolescent Psychiatry, 37(5), 512â€“ 518.
73. Hatcher, S., Sharon, C., Parag, V., & Collins, N. (2011). Problem-solving therapy for people who present to hospital with self-harm: Zelen randomised controlled trial. The British journal of psychiatry : the journal of mental science, 199(4), 310â€“316. https://doi.org/10.1192/bjp.bp.110.090126
74. Hatcher, S., Sharon, C., House, A., Collins, N., Collings, S., & Pillai, A. (2015). The ACCESS study: Zelen randomised controlled trial of a package of care for people presenting to hospital after self-harm. The British journal of psychiatry : the journal of mental science, 206(3), 229â€“236. https://doi.org/10.1192/bjp.bp.113.135780
75. Hatcher, S., Coupe, N., Wikiriwhi, K., Durie, S. M., & Pillai, A. (2016). Te Ira Tangata: a Zelen randomised controlled trial of a culturally informed treatment compared to treatment as usual in MÄori who present to hospital after selfharm. Social psychiatry and psychiatric epidemiology, 51(6), 885â€“894. https://doi.org/10.1007/s00127-016-1194-7
76. Hazell, P. L., Martin, G., Mcgill, K., Kay, T., Wood, A., Trainor, G., & Harrington, R. (2009). Group therapy for repeated deliberate self-harm in adolescents: failure of replication of a randomized trial. Journal of the American Academy of Child and Adolescent Psychiatry, 48(6), 662â€“670. https://doi.org/10.1097/CHI.0b013e3181aOacec
77. Herrmann, T. S., Nazarenko, E., Marchand, W., Day, A., Merrill, J., Neil, M., ... & Bryan, C. (2024). Randomized controlled trial of a brief mindfulness-based intervention for suicidal ideation among veterans. Military medicine, 189(3-4), 732-741.
78. Hetrick, S. E., Yuen, H. P., Bailey, E., Cox, G. R., Templer, K., Rice, S. M., Bendall, S., & Robinson, J. (2017). Internet-based cognitive behavioural therapy for young people with suicide-related behaviour (Reframe-IT): a randomised controlled trial. Evidence-based mental health, 20(3), 76â€“82. https://doi.org/10.1136/eb-2017-102719
79. Hill, R. M., & Pettit, J. W. (2019). Pilot Randomized Controlled Trial of LEAP: A Selective Preventive Intervention to Reduce Adolescents' Perceived Burdensomeness. Journal of clinical child and adolescent psychology : the official journal for the Society of Clinical Child and Adolescent Psychology, American Psychological Association, Division 53, 48(sup1), S45â€“S56. https://doi.org/10.1080/15374416.2016.1188705
80. HÃ¶gberg, G., & HÃ¤llstrÃ¶m, T. (2018). Mood Regulation Focused CBT Based on Memory Reconsolidation, Reduced Suicidal Ideation and Depression in Youth in a Randomised Controlled Study. International journal of environmental research and public health, 15(5), 921. https://doi.org/10.3390/ijerph15050921
81. Huey, S. J., Jr, Henggeler, S. W., Rowland, M. D., Halliday-Boykins, C. A., Cunningham, P. B., Pickrel, S. G., & Edwards, J. (2004). Multisystemic therapy effects on attempted suicide by youths presenting psychiatric emergencies. Journal of the American Academy of Child and Adolescent Psychiatry, 43(2), 183â€“190. https://doi.org/10.1097/00004583-200402000- 00014
82. Husain, N., Afsar, S., Ara, J., Fayyaz, H., Rahman, R. U., Tomenson, B., Hamirani, M., Chaudhry, N., Fatima, B., Husain, M., Naeem, F., & Chaudhry,B. (2014). Brief psychological intervention after self-harm: randomised controlled trial from Pakistan. The British journal of psychiatry : the journal of mental science, 204(6), 462â€“470. https://doi.org/10.1192/bjp.bp.113.138370
83. Husain, N., Kiran, T., Chaudhry, I. B., Williams, C., Emsley, R., Arshad, U., ... & Chaudhry, N. (2023). A culturally adapted manual-assisted problem-solving intervention (CMAP) for adults with a history of self-harm: a multi-centre randomised controlled trial. BMC medicine, 21(1), 282.
84. Hvid, M., Vangborg, K., SÃ¸rensen, H. J., Nielsen, I. K., Stenborg, J. M., & Wang, A. G. (2011). Preventing repetition of attempted suicide--II. The Amager project, a randomized controlled trial. Nordic journal of psychiatry, 65(5), 292â€“298. https://doi.org/10.3109/08039488.2010.544404
85. Ilgen, M. A., Olson-Madden, J. H., Price, A., Brenner, L. A., King, C. A., & Blow, F. C. (2023). Cognitive behavioral therapy for suicide prevention among Veterans receiving substance use disorder treatment: Results from a randomized trial.Â Journal of psychiatric research,Â 168, 344-352.
86. Interian, A., Chesin, M. S., Stanley, B., Latorre, M., Hill, L. M. S., Miller, R. B., ... & Kline, A. (2021). Mindfulness-based cognitive therapy for preventing suicide in military veterans: a randomized clinical trial.Â The Journal of Clinical Psychiatry,Â 82(5), 36479.
87. Jamshidi, F., Rajabi, S., & Dehghani, Y. (2021). How to heal their psychological wounds? effectiveness of EMDR therapy on postâ€traumatic stress symptoms, mindâ€wandering and suicidal ideation in Iranian child abuse victims.Â Counselling and Psychotherapy Research,Â 21(2), 412-421.
88. Jobes, D. A., Comtois, K. A., Gutierrez, P. M., Brenner, L. A., Huh, D., Chalker, S. A., Ruhe, G., Kerbrat, A. H., Atkins, D. C., Jennings, K., Crumlish, J., Corona, C. D., Connor, S. O., Hendricks, K. E., Schembari, B., Singer, B., & Crow, B. (2017). A Randomized Controlled Trial of the Collaborative Assessment and Management of Suicidality versus Enhanced Care as Usual With Suicidal Soldiers. Psychiatry, 80(4), 339â€“356. https://doi.org/10.1080/00332747.2017.1354607
89. Johnson, J. E., Stout, R. L., Miller, T. R., Zlotnick, C., Cerbo, L. A., Andrade,J. T., Nargiso, J., Bonner, J., & Wiltsey-Stirman, S. (2019). Randomized cost- effectiveness trial of group interpersonal psychotherapy (IPT) for prisoners with major depression. Journal of consulting and clinical psychology, 87(4), 392â€“406. https://doi.org/10.1037/ccp0000379
90. Kaslow, N. J., Leiner, A. S., Reviere, S., Jackson, E., Bethea, K., Bhaju, J., Rhodes, M., Gantt, M. J., Senter, H., & Thompson, M. P. (2010). Suicidal, abused African American women's response to a culturally informed intervention. Journal of consulting and clinical psychology, 78(4), 449â€“458. https://doi.org/10.1037/a0019692
91. King, C. A., Gipson, P. Y., Horwitz, A. G., & Opperman, K. J. (2015). Teen options for change: an intervention for young emergency patients who screen positive for suicide risk. Psychiatric services (Washington, D.C.), 66(1), 97â€“ 100. https://doi.org/10.1176/appi.ps.201300347
92. Koons, Cedar & Robins, Clive & Tweed, J. & Lynch, Thomas & Gonzalez, Alicia & Morse, Jennifer & Bishop, G. & Butterfield, Marian. (2001). Efficacy of dialectical behavior therapy in women veterans with borderline Personality disorder. Behavior Therapy. 32. 371-390. 10.1016/S0005- 7894(01)80009-5.
93. LaCroix, J. M., Perera, K. U., Neely, L. L., Grammer, G., Weaver, J., & Ghahramanlou-Holloway, M. (2018). Pilot trial of post-admission cognitive therapy: Inpatient program for suicide prevention. Psychological services, 15(3), 279â€“288. https://doi.org/10.1037/ser0000224 100.Smits, M. L., Feenstra, D. J., Eeren, H. V., Bales, D. L., Laurenssen, E.,
94. Liberman, R. P., & Eckman, T. (1981). Behavior therapy vs insight-oriented therapy for repeated suicide attempters. Archives of general psychiatry, 38(10), 1126â€“1130. https://doi.org/10.1001/archpsyc.1981.01780350060007
95. Lin, C. J., Huang, Y. H., Huang, K. Y., Wu, S. I., Chang, Y. H., Yeh, H. M., ... & Liu, S. I. (2020). A randomized controlled trial of transcultural validation of group-based psychosocial intervention for patients with bipolar disorder.Â Psychiatry Research,Â 290, 113139.
96. Lin, Y. C., Liu, S. I., Chen, S. C., Sun, F. J., Huang, H. C., Huang, C. R., & Chiu, Y. C. (2020). Brief cognitiveâ€based psychosocial intervention and case management for suicide attempters discharged from the emergency department in Taipei, taiwan: a randomized controlled study.Â Suicide and Lifeâ€Threatening Behavior,Â 50(3), 688-705.
97. Linehan, M. M., Comtois, K. A., Murray, A. M., Brown, M. Z., Gallop, R. J., Heard, H. L., Korslund, K. E., Tutek, D. A., Reynolds, S. K., & Lindenboim, N. (2006). Two-year randomized controlled trial and follow-up of dialectical behavior therapy vs therapy by experts for suicidal behaviors and borderline personality disorder. Archives of general psychiatry, 63(7), 757â€“766. https://doi.org/10.1001/archpsyc.63.7.757
98. LoParo, D., Mack, S. A., Patterson, B., Negi, L. T., & Kaslow, N. J. (2018). The efficacy of cognitively-based compassion training for African American suicide attempters. Mindfulness, 9(6), 1941â€“1954. https://doi.org/10.1007/s12671-018-0940-1
99. Lu, R., Zhou, Y., Wu, Q., Peng, X., Dong, J., Zhu, Z., & Xu, W. (2019). The effects of mindfulness training on suicide ideation among left-behind children in China: A randomized controlled trial. Child: care, health and development, 45(3), 371â€“379. https://doi.org/10.1111/cch.12650
100. Lynch, T. R., Hempel, R. J., Whalley, B., Byford, S., Chamba, R., Clarke, P., ... & Russell, I. T. (2020). Refractory depressionâ€“mechanisms and efficacy of radically open dialectical behaviour therapy (RefraMED): findings of a randomised trial on benefits and harms.Â The British Journal of Psychiatry,Â 216(4), 204-212.
101. Malakouti, S. K., Nojomi, M., Ghanbari, B., Rasouli, N., Khaleghparast, S., & Farahani, I. G. (2022). Aftercare and Suicide Reattempt Prevention in Tehran, Iran. Crisis, 43(1), 18-27.
102. Marasinghe, R. B., Edirippulige, S., Kavanagh, D., Smith, A., & Jiffry, M. T. (2012). Effect of mobile phone-based psychotherapy in suicide prevention: a randomized controlled trial in Sri Lanka. Journal of telemedicine and telecare, 18(3), 151â€“155. https://doi.org/10.1258/jtt.2012.SFT107
103. Marasinghe, R. B., Edirippulige, S., Kavanagh, D., Smith, A., & Jiffry, M. T. (2012). Effect of mobile phone-based psychotherapy in suicide prevention: a randomized controlled trial in Sri Lanka. Journal of telemedicine and telecare, 18(3), 151â€“155. https://doi.org/10.1258/jtt.2012.SFT107
104. March, J., Silva, S., Petrycki, S., Curry, J., Wells, K., Fairbank, J., Burns, B., Domino, M., McNulty, S., Vitiello, B., Severe, J., & Treatment for Adolescents With Depression Study (TADS) Team (2004). Fluoxetine, cognitive-behavioral therapy, and their combination for adolescents with depression: Treatment for Adolescents With Depression Study (TADS) randomized controlled trial. JAMA, 292(7), 807â€“820. https://doi.org/10.1001/jama.292.7.807
105. March, J., Silva, S., Petrycki, S., Curry, J., Wells, K., Fairbank, J., Burns, B., Domino, M., McNulty, S., Vitiello, B., Severe, J., & Treatment for Adolescents With Depression Study (TADS) Team (2004). Fluoxetine, cognitive-behavioral therapy, and their combination for adolescents with depression: Treatment for Adolescents With Depression Study (TADS) randomized controlled trial. JAMA, 292(7), 807â€“820. https://doi.org/10.1001/jama.292.7.807
106. McAuliffe, C., McLeavey, B. C., Fitzgerald, T., Corcoran, P., Carroll, B., Ryan, L., O'Keeffe, B., Fitzgerald, E., Hickey, P., O'Regan, M., Mulqueen, J., & Arensman, E. (2014). Group problem-solving skills training for self-harm: randomised controlled trial. The British journal of psychiatry : the journal of mental science, 204, 383â€“390. https://doi.org/10.1192/bjp.bp.111.101816
107. McCauley, E., Berk, M. S., Asarnow, J. R., Adrian, M., Cohen, J., Korslund, K., Avina, C., Hughes, J., Harned, M., Gallop, R., & Linehan, M. M. (2018). Efficacy of Dialectical Behavior Therapy for Adolescents at High Risk for Suicide: A Randomized Clinical Trial. JAMA psychiatry, 75(8), 777â€“785. https://doi.org/10.1001/jamapsychiatry.2018.1109
108. McLeavey, B. C., Daly, R. J., Ludgate, J. W., & Murray, C. M. (1994). Interpersonal Problemâ€Solving skills training in the treatment of selfâ€poisoning patients.Â Suicide and Lifeâ€Threatening Behavior,Â 24(4), 382-394.
109. McMain, S. F., Guimond, T., Barnhart, R., Habinski, L., & Streiner, D. L. (2017). A randomized trial of brief dialectical behaviour therapy skills training in suicidal patients suffering from borderline disorder. Acta psychiatrica Scandinavica, 135(2), 138â€“148. https://doi.org/10.1111/acps.12664
110. McManama O'Brien, K. H., Sellers, C. M., Battalen, A. W., Ryan, C. A., Maneta, E. K., Aguinaldo, L. D., White, E., & Spirito, A. (2018). Feasibility, acceptability, and preliminary effects of a brief alcohol intervention for suicidal adolescents in inpatient psychiatric treatment. Journal of substance abuse treatment, 94, 105â€“112. https://doi.org/10.1016/j.jsat.2018.08.013
111. Melvin, G. A., Tonge, B. J., King, N. J., Heyne, D., Gordon, M. S., & Klimkeit, E. (2006). A comparison of cognitive-behavioral therapy, sertraline, and their combination for adolescent depression. Journal of the American Academy of Child and Adolescent Psychiatry, 45(10), 1151â€“1161. https://doi.org/10.1097/01.chi.0000233157.21925.71
112. Miklowitz, D. J., Merranko, J. A., Weintraub, M. J., Walshaw, P. D., Singh, M. K., Chang, K. D., & Schneck, C. D. (2020). Effects of family-focused therapy on suicidal ideation and behavior in youth at high risk for bipolar disorder.Â Journal of affective disorders,Â 275, 14-22.
113. MontesÃ³-Curto, P., GarcÃ­a-MartÃ­nez, M., GÃ³mez-MartÃ­nez, C., FerrÃ©-Almo, S., Panisello-Chavarria, M. L., GenÃ­s, S. R., Mateu Gil, M. L., CubÃ­ GuillÃ©n, M. T., ColÃ¡s, L. S., Usach, T. S., Herrero, A. S., & FerrÃ©-Grau, C. (2015).
114. Morley, K. C., Sitharthan, G., Haber, P. S., Tucker, P., & Sitharthan, T. (2014). The efficacy of an opportunistic cognitive behavioral intervention package (OCB) on substance use and comorbid suicide risk: a multisite randomized controlled trial. Journal of consulting and clinical psychology, 82(1), 130â€“140. https://doi.org/10.1037/a0035310
115. Morthorst, B., Krogh, J., Erlangsen, A., Alberdi, F., & Nordentoft, M. (2012).Effect of assertive outreach after suicide attempt in the AID (assertive intervention for deliberate self harm) trial: randomised controlled trial. BMJ (Clinical research ed.), 345, e4972. https://doi.org/10.1136/bmj.e4972
116. SS Naidoo, P Gathiram & L Schlebusch (2014). Effectiveness of a Buddy intervention support programme for suicidal behaviour in a primary care setting, South African Family Practice, 56:5, 263-270, DOI: 10.1080/20786190.2014.980159
117. Nakimuli-Mpungu, E., Musisi, S., Wamala, K., Okello, J., Ndyanabangi, S., Birungi, J., ... & Mills, E. J. (2020). Effectiveness and cost-effectiveness of group support psychotherapy delivered by trained lay health workers for depression treatment among people with HIV in Uganda: a cluster-randomised trial.Â The Lancet Global Health,Â 8(3), e387-e398.
118. O'Connor, R. C., Ferguson, E., Scott, F., Smyth, R., McDaid, D., Park, A. L., Beautrais, A., & Armitage, C. J. (2017). A brief psychological intervention to reduce repetition of self-harm in patients admitted to hospital following a suicide attempt: a randomised controlled trial. The lancet. Psychiatry, 4(6), 451â€“460. https://doi.org/10.1016/S2215-0366(17)30129-3
119. O'Toole, M. S., Arendt, M. B., & Pedersen, C. M. (2019). Testing an App- Assisted Treatment for Suicide Prevention in a Randomized Controlled Trial: Effects on Suicide Risk and Depression. Behavior therapy, 50(2), 421â€“429. https://doi.org/10.1016/j.beth.2018.07.007
120. Pachankis, J. E., Harkness, A., Maciejewski, K. R., Behari, K., Clark, K. A., McConocha, E., ... & Safren, S. A. (2022). LGBQ-affirmative cognitive-behavioral therapy for young gay and bisexual menâ€™s mental and sexual health: A three-arm randomized controlled trial.Â Journal of consulting and clinical psychology,Â 90(6), 459.
121. Patel, V., Weobong, B., Weiss, H. A., Anand, A., Bhat, B., Katti, B., Dimidjian, S., Araya, R., Hollon, S. D., King, M., Vijayakumar, L., Park, A. L., McDaid, D., Wilson, T., Velleman, R., Kirkwood, B. R., & Fairburn, C. G. (2017). The Healthy Activity Program (HAP), a lay counsellor-delivered brief psychological treatment for severe depression, in primary care in India: a randomised controlled trial. Lancet (London, England), 389(10065), 176â€“185. https://doi.org/10.1016/S0140-6736(16)31589-6
122. Patsiokas, A. T., & Clum, G. A. (1985). Effects of psychotherapeutic strategies in the treatment of suicide attempters. Psychotherapy: Theory, Research, Practice, Training, 22(2), 281â€“290. https://doi.org/10.1037/h0085507
123. Peters, E., Landau, S., McCrone, P., Cooke, M., Fisher, P., Steel, C., Evans, R., Carswell, K., Dawson, K., Williams, S., Howard, A., & Kuipers, E. (2010). A randomised controlled trial of cognitive behaviour therapy for psychosis in a routine clinical service. Acta psychiatrica Scandinavica, 122(4), 302â€“318. https://doi.org/10.1111/j.1600-0447.2010.01572.x
124. Pfeiffer, P. N., King, C., Ilgen, M., Ganoczy, D., Clive, R., Garlick, J., Abraham, K., Kim, H. M., Vega, E., Ahmedani, B., & Valenstein, M. (2019). Development and pilot study of a suicide prevention intervention delivered by peer support specialists. Psychological services, 16(3), 360â€“371. https://doi.org/10.1037/ser0000257
125. Pigeon, W. R., Funderburk, J., Bishop, T. M., & Crean, H. F. (2017). Brief cognitive behavioral therapy for insomnia delivered to depressed veterans receiving primary care services: A pilot study. Journal of affective disorders, 217, 105â€“111. https://doi.org/10.1016/j.jad.2017.04.003
126. Pigeon, W. R., Funderburk, J. S., Cross, W., Bishop, T. M., & Crean, H. F. (2019). Brief CBT for insomnia delivered in primary care to patients endorsing suicidal ideation: a proof-of-concept randomized clinical trial. Translational behavioral medicine, 9(6), 1169â€“1177. https://doi.org/10.1093/tbm/ibz108
127. Pineda, J., & Dadds, M. R. (2013). Family intervention for adolescents with suicidal behavior: a randomized controlled trial and mediation analysis. Journal of the American Academy of Child and Adolescent Psychiatry, 52(8), 851â€“862. https://doi.org/10.1016/j.jaac.2013.05.015
128. Pistorello, J., Jobes, D. A., Gallop, R., Compton, S. N., Locey, N. S., Au, J. S., Noose, S. K., Walloch, J. C., Johnson, J., Young, M., Dickens, Y., Chatham, P., & Jeffcoat, T. (2021). A Randomized Controlled Trial of the Collaborative Assessment and Management of Suicidality (CAMS) Versus Treatment as Usual (TAU) for Suicidal College Students. Archives of suicide research : official journal of the International Academy for Suicide Research, 25(4), 765â€“789. https://doi.org/10.1080/13811118.2020.1749742
129. Power, P. J., Bell, R. J., Mills, R., Herrman-Doig, T., Davern, M., Henry, L., Yuen, H. P., Khademy-Deljo, A., & McGorry, P. D. (2003). Suicide prevention in first episode psychosis: the development of a randomised controlled trial of cognitive therapy for acutely suicidal patients with early psychosis. The Australian and New Zealand journal of psychiatry, 37(4), 414â€“ 420. https://doi.org/10.1046/j.1440-1614.2003.01209.x
130. Pratt, D., Tarrier, N., Dunn, G., Awenat, Y., Shaw, J., Ulph, F., & Gooding, P. (2015). Cognitive-behavioural suicide prevention for male prisoners: a pilot randomized controlled trial. Psychological medicine, 45(16), 3441â€“3451. https://doi.org/10.1017/S0033291715001348
131. Rahnama, Shirin & Tarkhan, Morteza & Khalatbari, Javad. (2013). Effectiveness of Imagery Rescripting and Reprocessing Therapy on Suicidal Ideation in Individuals with Suicide Attempt History. Procedia - Social and Behavioral Sciences. 84. 10.1016/j.sbspro.2013.06.706.
132. Robinson, W. L., Whipple, C. R., Keenan, K., Flack, C. E., Lemke, S., & Jason, L. A. (2023). Reducing suicidal ideation in African American adolescents: A randomized controlled clinical trial.Â Journal of consulting and clinical psychology.
133. Rossouw, T. I., & Fonagy, P. (2012). Mentalization-based treatment for self- harm in adolescents: a randomized controlled trial. Journal of the American Academy of Child and Adolescent Psychiatry, 51(12), 1304â€“1313.e3. https://doi.org/10.1016/j.jaac.2012.09.018
134. Rudd, M. D., Rajab, M. H., Orman, D. T., Joiner, T., Stulman, D. A., & Dixon, W. (1996). Effectiveness of an outpatient intervention targeting suicidal young adults: preliminary results. Journal of consulting and clinical psychology, 64(1), 179â€“190. https://doi.org/10.1037//0022-006x.64.1.179
135. Rudd, M. D., Bryan, C. J., Wertenberger, E. G., Peterson, A. L., Young- McCaughan, S., Mintz, J., Williams, S. R., Arne, K. A., Breitbach, J., Delano, K., Wilkinson, E., & Bruce, T. O. (2015). Brief cognitive-behavioral therapy effects on post-treatment suicide attempts in a military sample: results of a randomized clinical trial with 2-year follow-up. The American journal of psychiatry, 172(5), 441â€“449. https://doi.org/10.1176/appi.ajp.2014.14070843
136. Ryberg, W., Zahl, P. H., Diep, L. M., LandrÃ¸, N. I., & Fosse, R. (2019). Managing suicidality within specialized care: A randomized controlled trial. Journal of affective disorders, 249, 112â€“120. https://doi.org/10.1016/j.jad.2019.02.022
137. Salkovskis, P. M., Atha, C., & Storer, D. (1990). Cognitive-behavioural problem solving in the treatment of patients who repeatedly attempt suicide a controlled trial. The British Journal of Psychiatry, 157(6), 871-876.
138. Samaraweera, S., Sivayogan, S., Sumathipala, A., Bhugra, D., & Siribaddana,S. (2007). RCT of Cognitive Behaviour Therapy in active suicidal ideation-as feasibility study in Sri Lanka. The European Journal of Psychiatry, 21(3), 175â€“178. https://doi.org/10.4321/S0213-61632007000300001
139. Sanz Cruces, J. M., GarcÃ­a Cuenca, I. M., Lacomba-Trejo, L., Cuquerella Adell, M. Ã., Cano Navarro, I., Ferrandis CortÃ©s, M., JordÃ¡ Carreres, E., & Carbajo Ãlvarez, E. (2018). Group Therapy for Patients with Adjustment Disorder in Primary Care. The Spanish journal of psychology, 21, E50. https://doi.org/10.1017/sjp.2018.51
140. Sedghy, Z., Yoosefi, N., & Navidian, A. (2020). The effect of motivational interviewing-based training on the rate of using mental health services and intensity of suicidal ideation in individuals with suicide attempt admitted to the emergency department.Â Journal of education and health promotion,Â 9.
141. Shaygan, M., Sheybani Negad, S., & Motazedian, S. (2022). The effect of combined sertraline and positive psychotherapy on hopelessness and suicidal ideation among patients with major depressive disorder: a randomized controlled trial.Â The Journal of Positive Psychology,Â 17(5), 655-664.
142. Sheaves, B., Holmes, E. A., Rek, S., Taylor, K. M., Nickless, A., Waite, F., Germain, A., Espie, C. A., Harrison, P. J., Foster, R., & Freeman, D. (2019). Cognitive Behavioural Therapy for Nightmares for Patients with Persecutory Delusions (Nites): An Assessor-Blind, Pilot Randomized Controlled Trial. Canadian journal of psychiatry. Revue canadienne de psychiatrie, 64(10), 686â€“696. https://doi.org/10.1177/0706743719847422
143. Simpson, G. K., Tate, R. L., Whiting, D. L., & Cotter, R. E. (2011). Suicide prevention after traumatic brain injury: a randomized controlled trial of a program for the psychological treatment of hopelessness. The Journal of head trauma rehabilitation, 26(4), 290â€“300.
144. Sinniah, A., Oei, T., Maniam, T., & Subramaniam, P. (2017). Positive effects of Individual Cognitive Behavior Therapy for patients with unipolar mood disorders with suicidal ideation in Malaysia: A randomised controlled trial. Psychiatry research, 254, 179â€“189. https://doi.org/10.1016/j.psychres.2017.04.026
145. Slee, N., Garnefski, N., van der Leeden, R., Arensman, E., & Spinhoven, P. (2008). Cognitive-behavioural intervention for self-harm: randomised controlled trial. The British journal of psychiatry : the journal of mental science, 192(3), 202â€“211. https://doi.org/10.1192/bjp.bp.107.037564
146. Slesnick, N., Zhang, J., Feng, X., Wu, Q., Walsh, L., & Granello, D. H. (2020). Cognitive therapy for suicide prevention: A randomized pilot with suicidal youth experiencing homelessness.Â Cognitive Therapy and Research,Â 44, 402-411.
147. Spirito, A., Wolff, J. C., Seaboyer, L. M., Hunt, J., Esposito-Smythers, C., Nugent, N., Zlotnick, C., & Miller, I. (2015). Concurrent treatment for adolescent and parent depressed mood and suicidality: feasibility, acceptability, and preliminary findings. Journal of child and adolescent psychopharmacology, 25(2), 131â€“139. https://doi.org/10.1089/cap.2013.0130
148. Springer, T., Lohr, N. E., Buchtel, H. A., & Silk, K. R. (1996). A preliminary report of short-term cognitive-behavioral group therapy for inpatients with personality disorders. The Journal of psychotherapy practice and research, 5(1), 57â€“71.
149. Tang, T. C., Jou, S. H., Ko, C. H., Huang, S. Y., & Yen, C. F. (2009). Randomized study of school-based intensive interpersonal psychotherapy for depressed adolescents with suicidal risk and parasuicide behaviors. Psychiatry and clinical neurosciences, 63(4), 463â€“470. https://doi.org/10.1111/j.1440- 1819.2009.01991.x
150. Tarrier, N., Kelly, J., Maqsood, S., Snelson, N., Maxwell, J., Law, H., Dunn, G., & Gooding, P. (2014). The cognitive behavioural prevention of suicide in psychosis: a clinical trial. Schizophrenia research, 156(2-3), 204â€“210. https://doi.org/10.1016/j.schres.2014.04.029
151. Tighe, J., Shand, F., Ridani, R., Mackinnon, A., De La Mata, N., & Christensen, H. (2017). Ibobbly mobile health intervention for suicide prevention in Australian Indigenous youth: a pilot randomised controlled trial. BMJ open, 7(1), e013518. https://doi.org/10.1136/bmjopen-2016-013518 174.Turner, R. M. (2000). Naturalistic evaluation of dialectical behavior therapy-oriented treatment for borderline personality disorder. Cognitive and Behavioral Practice, 7(4), 413â€“419. https://doi.org/10.1016/S1077-7229(00)80052-8
152. Turner, R. M. (2000). Naturalistic evaluation of dialectical behavior therapyoriented treatment for borderline personality disorder. Cognitive and Behavioral Practice, 7(4), 413â€“419. https://doi.org/10.1016/S1077- 7229(00)80052-8
153. Tyrer, P., Thompson, S., Schmidt, U., Jones, V., Knapp, M., Davidson, K., Catalan, J., Airlie, J., Baxter, S., Byford, S., Byrne, G., Cameron, S., Caplan, R., Cooper, S., Ferguson, B., Freeman, C., Frost, S., Godley, J., Greenshields, J., Henderson, J., â€¦ Wessely, S. (2003). Randomized controlled trial of brief cognitive behaviour therapy versus treatment as usual in recurrent deliberate self-harm: the POPMACT study. Psychological medicine, 33(6), 969â€“976. https://doi.org/10.1017/s0033291703008171
154. Van Beek, W. Future thinking in suicidal patients, Ede: Print Service Ede B.V. , 2013, p226.
155. van der Sande, R., van Rooijen, L., Buskens, E., Allart, E., Hawton, K., van der Graaf, Y., & van Engeland, H. (1997). Intensive in-patient and community intervention versus routine care after attempted suicide. A randomised controlled intervention study. The British journal of psychiatry : the journal of mental science, 171, 35â€“41. https://doi.org/10.1192/bjp.171.1.35
156. Van Orden, K. A., AreÃ¡n, P. A., & Conwell, Y. (2021). A pilot randomized trial of engage psychotherapy to increase social connection and reduce suicide risk in later life.Â The American Journal of Geriatric Psychiatry,Â 29(8), 789-800.
157. van Wel, E. B., Bos, E. H., Appelo, M. T., Berendsen, E. M., Willgeroth, F. C., & Verbraak, M. J. (2009). De effectiviteit van de vaardigheidstraining emotieregulatiestoornis (VERS) in de behandeling van de borderlinepersoonlijkheidsstoornis; een gerandomiseerd onderzoek [The efficacy of the systems training for emotional predictability and problem solving (STEPPS) in the treatment of borderline personality disorder. A randomized controlled trial]. Tijdschrift voor psychiatrie, 51(5), 291â€“301.
158. Verheul, R., Van Den Bosch, L. M., Koeter, M. W., De Ridder, M. A., Stijnen, T., & Van Den Brink, W. (2003). Dialectical behaviour therapy for women with borderline personality disorder: 12-month, randomised clinical trial in The Netherlands. The British journal of psychiatry : the journal of mental science, 182, 135â€“140. https://doi.org/10.1192/bjp.182.2.135
159. Walker, T., Shaw, J., Turpin, C., Reid, C., & Abel, K. (2017). The WORSHIP II study: a pilot of psychodynamic interpersonal therapy with women offenders who self-harm.Â The Journal of Forensic Psychiatry & Psychology,Â 28(2), 158-171.
160. Waraan, L., Rognli, E. W., Czajkowski, N. O., Mehlum, L., & Aalberg, M. (2021). Efficacy of attachment-based family therapy compared to treatment as usual for suicidal ideation in adolescents with MDD.Â Clinical child psychology and psychiatry,Â 26(2), 464â€“474. https://doi.org/10.1177/1359104520980776
161. Ward-Ciesielski, E. F., Tidik, J. A., Edwards, A. J., & Linehan, M. M. (2017).Comparing brief interventions for suicidal individuals not engaged in treatment: A randomized clinical trial. Journal of affective disorders, 222, 153â€“161. https://doi.org/10.1016/j.jad.2017.07.011
162. Wei, S., Liu, L., Bi, B., Li, H., Hou, J., Tan, S., Chen, X., Chen, W., Jia, X.,Dong, G., Qin, X., & Liu, Y. (2013). An intervention and follow-up study following a suicide attempt in the emergency departments of four general hospitals in Shenyang, China. Crisis, 34(2), 107â€“115. https://doi.org/10.1027/0227-5910/a000181
163. Weinberg, I., Gunderson, J. G., Hennen, J., & Cutter, C. J., Jr (2006). Manual assisted cognitive treatment for deliberate self-harm in borderline personality disorder patients. Journal of personality disorders, 20(5), 482â€“492. https://doi.org/10.1521/pedi.2006.20.5.482
164. Weinstein, S. M., Cruz, R. A., Isaia, A. R., Peters, A. T., & West, A. E. (2018). Child- and Family-Focused Cognitive Behavioral Therapy for Pediatric Bipolar Disorder: Applications for Suicide Prevention. Suicide & life-threatening behavior, 48(6), 797â€“811. https://doi.org/10.1111/sltb.12416
165. Wharff, E. A., Ginnis, K. B., Ross, A. M., White, E. M., White, M. T., & Forbes, P. W. (2019). Family-Based Crisis Intervention With Suicidal Adolescents: A Randomized Clinical Trial. Pediatric emergency care, 35(3), 170â€“175. https://doi.org/10.1097/PEC.0000000000001076
166. Wilks, C. R., Lungu, A., Ang, S. Y., Matsumiya, B., Yin, Q., & Linehan, M.M. (2018). A randomized controlled trial of an Internet delivered dialectical behavior therapy skills training for suicidal and heavy episodic drinkers. Journal of affective disorders, 232, 219â€“228. https://doi.org/10.1016/j.jad.2018.02.053
167. Wood, A., Trainor, G., Rothwell, J., Moore, A., & Harrington, R. (2001). Randomized trial of group therapy for repeated deliberate self-harm in adolescents. Journal of the American Academy of Child and Adolescent Psychiatry, 40(11), 1246â€“1253. https://doi.org/10.1097/00004583-200111000-
168. Wu, R., Zhong, S. Y., Wang, G. H., Wu, M. Y., Xu, J. F., Zhu, H., ... & Jiang, C. L. (2023). The effect of brief mindfulness meditation on suicidal ideation, stress and sleep quality.Â Archives of suicide research,Â 27(2), 215-230.
169. Wu, S. I., Huang, H. C., Chen, J. S. C., Korslund, K., Lin, C. J., Lin, Y., ... & Liu, S. I. (2023). Dialectical Behavior Therapy in suicidal patients with borderline personality disorder: A pilot randomized controlled trial in Taiwan.
170. Xie, Z., Li, X., Yin, J., Wang, M., Fan, X., Yu, J., ... & Yu, M. (2023). Value of Group Intervention on Prognosis of Quality of Life in Epileptic Patients Treated With Sodium Valproate and Lamotrigine.Â Alternative Therapies in Health & Medicine,Â 29(3).
171. Yen, S., Ranney, M. L., Krek, M., Peters, J. R., Mereish, E. H., Tezanos, K. M., Kahler, C. W., Solomon, J., Beard, C., & Spirito, A. (2020). Skills to Enhance Positivity in Suicidal Adolescents: Results from a Pilot Randomized Clinical Trial. The journal of positive psychology, 15(3), 348â€“361. https://doi.org/10.1080/17439760.2019.1615105
172. Zhao, J., Chen, D. Y., Li, X. B., Xi, Y. J., Verma, S., Zhou, F. C., & Wang, C. Y. (2023). EMDR versus waiting list in individuals at clinical high risk for psychosis with post-traumatic stress symptoms: A randomized controlled trial.Â Schizophrenia research,Â 256, 1-7.

## Appendix C: Characteristics of included studies

| Study | Intervention | Control group | Country | Suicide as incl. criterium | Recruitment | Prop. women | Age group | Setting | Format | Risk of Bias | |
| --- | --- | --- | --- | --- | --- | --- | --- | --- | --- | --- | --- |
| Alavi 2013 | cbt | wl | oth | yes | oth | 0.90 | adol | outpatient | individual and family-based | high | |
| Amianto 2011 | dyn | cau | eu | no | clin | 0.48 | adult | outpatient | individual | some concerns | |
| Andreoli 2016 | dyn | cau | eu | yes | clin | 0.81 | adult | outpatient | individual | high | |
| Ardashir 2018 | cbt | cau | oth | yes | clin | 0.50 | adult | inpatient | group | high | |
| Arnevik 2009 | other | ecau | eu | no | clin | 0.74 | adult | outpatient | group | high | |
| Arvilommi 2022 | assip | supp | eu | yes | clin | 0.71 | adult | outpatient | individual | high | |
| Asarnow 2011 | fam | ecau | usa | yes | oth | 0.69 | adol | inpatient | family-based | high | |
| Asarnow 2017 | fam | ecau | usa | yes | clin | 0.88 | adol | mixed | family-based | low | |
| Asarnow 2019 | fam | supp | usa | no | clin | 0.56 | child | outpatient | family-based | some concerns | |
| Ayar 2020 | pst | cau | oth | no | clin | 0.48 | adult | inpatient | individual | high | |
| Bannan 2010 | pst | cau | eu | yes | oth | 1 | adult | outpatient | group | high | |
| Barnes 2021 | other | cau | usa | yes | clin | 0.19 | adult | inpatient | individual and group | high | |
| Barnhofer 2009 | mbct | wl | uk | yes | oth | 0.68 | adult | outpatient | group | high | |
| Bateman 1999 | dyn | cau | uk | no | clin | 0.57 | adult | inpatient | individual and group | NA | |
| Bateman 2009 | mbt | ecau | uk | yes | clin | 0.80 | adult | outpatient | individual and group | NA | |
| Blomdahl 2018 | other | cau | eu | no | oth | 0.71 | adult | outpatient | individual | high | |
| Brenner 2018 | cbt | wl | au | no | com | 0.57 | adult | outpatient | group | high | |
| Brent 1997 | cbt | supp | usa | no | clin | 0.76 | adol | outpatient | individual and family-based | high | |
| Britton 2020 | dyn | cau | usa | yes | clin | 0.11 | adult | inpatient | individual | high | |
| Brown 2005 | cbt | ecau | usa | yes | oth | 0.61 | adult | outpatient | individual | high | |
| Brown 2019 study 1 | img | wl | usa | no | oth | 0.11 | adult | outpatient | individual | high | |
| Brown 2019 study 2 | img | supp | usa | no | oth | 0.11 | adult | outpatient | individual | high | |
| Bryan 2017 | spi | ecau | usa | yes | clin | 0.22 | adult | inpatient | individual | some concerns | |
| Bryant 2024a | other | ecau | au | no | com | 0.84 | adult | outpatient | group | high | |
| Bryant 2024b | other | ecau | au | no | com | 0.84 | adult | outpatient | group | high | |
| Celano 2017 | mea | other ctr | usa | yes | clin | 0.69 | adult | outpatient | individual | high | |
| Chan 2022 | cbt | wl | eas | no | com | 0.67 | yadult | outpatient | group | high | |
| Chanen 2022 | other | ecau | au | no | clin | 0.81 | yadult | outpatient | individual | high | |
| Chen 2015 | other | other ctr | eas | no | clin | 0.73 | adult | outpatient | group | high | |
| Christopher 2024 | other | other ctr | us | no | com | 0.29 | adult | outpatient | group | high | |
| Comtois 2011 | cams | cau | usa | yes | clin | 0.62 | adult | outpatient | individual | high | |
| Conner 2021 | assip | cau | usa | no | clin | 0.65 | adult | inpatient | individual | high | |
| Cottraux 2009 | cbt | supp | eu | no | clin | 0.80 | adult | outpatient | individual | NA | |
| Cottrell 2018 | fam | cau | uk | yes | clin | 0.89 | adol | outpatient | family-based | high | |
| Crawford 2020 | mixed | cau | uk | no | clin | 0.68 | adult | outpatient | individual | high | |
| Daneshvar 2022 | other | wl | oth | no | clin | 1 | adult | outpatient | group | high | |
| Davidson 2006 | cbt | cau | uk | no | clin | NA | adult | outpatient | individual | low | |
| Davidson 2014 | cbt | cau | uk | yes | oth | NA | adult | outpatient | individual | high | |
| De Jonge-Heesen 2020 | cbt | other ctr | eu | no | com | 0.64 | adol | outpatient | group | NA | |
| De Groot 2007 | cbt | cau | eu | no | clin | NA | adult | outpatient | family-based | high | |
| De Jaegere 2023 | cbt | cau | eu | yes | com | 0.65 | adult | outpatient | group | high | |
| Diamond 2010 | fam | ecau | usa | yes | clin | 0.83 | adol | outpatient | family-based | high | |
| Diamond 2019 | fam | supp | usa | yes | oth | 0.82 | adol | outpatient | family-based | high | |
| Doering 2010 | dyn | ecau | eu | no | clin | 1 | adult | outpatient | individual | NA | |
| Donaldson 2005 | other | supp | usa | yes | clin | 0.82 | adol | outpatient | individual | high | |
| Ducasse 2018 | other | other ctr | eu | yes | clin | 0.88 | adult | outpatient | group | high | |
| Ducasse 2019 | other | other ctr | eu | yes | clin | 0.65 | adult | inpatient | individual | some concerns | |
| Ertl 2011 | img | wl | oth | no | com | 0.55 | yadult | outpatient | individual | high | |
| Eskin 2008 | pst | wl | oth | no | com | 0.70 | yadult | outpatient | individual | high | |
| Esposito-Smythers 2011 | cbt | cau | usa | yes | clin | 0.76 | adol | outpatient | individual and family-based | high | |
| Esposito-Smythers 2017 | cbt | wl | usa | no | clin | 0.67 | adol | outpatient | family-based | high | |
| Esposito-Smythers 2019 | fam | ecau | usa | yes | oth | 0.58 | adol | outpatient | family-based | high | |
| Evans 1999 | cbt | cau | uk | no | clin | NA | adult | outpatient | individual | high | |
| Eylem 2021 | cbt | wl | oth | yes | com | 0.72 | adult | outpatient | individual | high | |
| Fardig 2011 | other | cau | eu | no | clin | 0.46 | adult | outpatient | group | high | |
| Fereidouni 2019 | img | cau | oth | no | clin | 0.67 | adult | inpatient | individual | high | |
| Ghahramanlou-Holloway 2018 | cbt | cau | usa | yes | oth | 0.42 | adult | inpatient | individual | high | |
| Goldstein 2015 | dbt | cau | usa | no | NA | 0.75 | adol | outpatient | individual and family-based | high | |
| Goldstein 2023 | dbt | ecau | us | no | clin | 0.85 | adol | outpatient | individual and family-based | NA | |
| Goldston 2022 | cbt | cau | usa | no | clin | 0.62 | adol | outpatient | individual and family-based | high | |
| Goodman 2016 | dbt | cau | usa | yes | clin | 0.33 | adult | outpatient | individual and group | high | |
| Goodman 2022 | fam | cau | usa | yes | clin | 0.15 | adult | outpatient | family-based | high | |
| Goodyer 2008 | cbt | ecau | uk | no | clin | 0.74 | adol | outpatient | individual | high | |
| Green 2011 | other | cau | uk | yes | clin | 0.79 | adol | NA | group | high | |
| Gregory 2008 | dyn | cau | usa | no | clin | 0.80 | adult | outpatient | individual | NA | |
| Grupp-Phelan 2019 | other | ecau | usa | yes | oth | 0.79 | adol | outpatient | individual | high | |
| Guthrie 2001 | dyn | cau | uk | yes | oth | 0.56 | adult | outpatient | individual | high | |
| Gutierrez 2021 | cams | cau | usa | yes | clin | 0.17 | adult | outpatient | group | high | |
| Gysin-Maillart 2016 | assip | other ctr | eu | yes | oth | 0.55 | adult | outpatient | individual | some concerns | |
| Haddock 2019 | cbt | cau | uk | yes | clin | 0.57 | adult | inpatient | individual | high | |
| Hahm 2019 | other | wl | usa | no | com | 1 | yadult | outpatient | group | high | |
| Harrington 1998 | fam | cau | uk | yes | clin | 0.90 | adol | outpatient | family-based | high | |
| Hatcher 2011 | pst | cau | au | yes | oth | 0.69 | adult | outpatient | individual | high | |
| Hatcher 2015 | pst | cau | au | yes | oth | 0.68 | adult | outpatient | individual | NA | |
| Hatcher 2016 | pst | cau | au | yes | oth | 0.65 | adult | outpatient | individual | high | |
| Hazell 2009 | other | cau | au | yes | NA | 0.90 | adol | outpatient | group | high | |
| Hermann 2024 | other | cau | us | yes | clin | 0.20 | adult | inpatient | individual | high | |
| Hetrick 2017 | cbt | cau | au | yes | oth | 0.82 | adol | mixed | individual | high | |
| Hill 2019 | other | cau | usa | yes | com | 0.69 | adol | NA | individual | some concerns | |
| Hogberg 2018 | cbt | cau | eu | no | clin | 0.70 | adol | outpatient | individual | high | |
| Huey 2004 | fam | ecau | usa | yes | oth | 0.35 | child | inpatient | family-based | high | |
| Husain 2014 | pst | cau | oth | yes | oth | 0.69 | yadult | outpatient | individual | high | |
| Husain 2023 | pst | cau | other | yes | clin | 0.60 | adult | outpatient | individual and family-based | high | |
| Hvid 2011 | other | cau | eu | yes | oth | 0.71 | adult | outpatient | individual | NA | |
| Ilgen 2023 | cbt | other ctr | us | yes | clin | 0.10 | adult | outpatient | individual | NA | |
| Interian 2021 | mbct | ecau | usa | yes | clin | 0.12 | adult | mixed | individual and group | some concerns | |
| Jamshidi 2020 | img | wl | oth | no | oth | 1 | yadult | NA | individual | high | |
| Jobes 2017 | cams | cau | usa | yes | clin | 0.20 | adult | outpatient | individual | high | |
| Johnson (J) 2019 | ipt | cau | usa | no | oth | 0.35 | adult | outpatient | group | low | |
| Kaslow 2010 | other | cau | usa | yes | clin | 1 | adult | outpatient | group | high | |
| King 2015 | other | cau | usa | yes | oth | 0.80 | adol | outpatient | individual | high | |
| Koons 2001 | dbt | cau | usa | no | clin | 1 | adult | outpatient | individual and group | high | |
| LaCroix 2018 | cbt | cau | usa | yes | clin | 0.31 | adult | inpatient | individual | some concerns | |
| Liberman 1981 | dyn | mixed | usa | no | clin | 0.67 | adult | inpatient | individual and family-based | high | |
| Lin (C-J) 2020 | cbt | cau | eas | no | clin | 0.63 | adult | outpatient | group | high | |
| Lin (Y-C) 2020 | cbt | cau | eas | yes | clin | 0.72 | adult | outpatient | individual | high | |
| Linehan 2006 | dbt | ecau | usa | yes | clin | 1 | adult | outpatient | individual and group | high | |
| LoParo 2018 | cbt | other ctr | usa | yes | clin | 0.53 | adult | outpatient | group | high | |
| Lu 2019 | mbct | wl | eas | no | oth | 0.25 | child | outpatient | group | high | |
| Lynch 2020 | dbt | cau | uk | no | clin | 0.66 | NA | outpatient | individual | high | |
| Malakouti 2022 | other | cau | oth | yes | clin | 0.67 | adult | outpatient | individual | high | |
| Marasinghe 2012 men | other | wl | oth | yes | clin | 0 | adult | mixed | individual | high | |
| Marasinghe 2012 women | mixed | wl | oth | yes | clin | 1 | adult | mixed | individual | high | |
| March 2004 study 1 | cbt | ecau | usa | no | com | 0.54 | adol | outpatient | individual | high | |
| March 2004 study 2 | cbt | placebo | usa | no | com | 0.54 | adol | outpatient | individual | high | |
| McAuliffe 2014 | pst | cau | eu | yes | oth | 0.64 | adult | outpatient | group | high | |
| McCauley 2018 | dbt | supp | usa | yes | oth | 0.95 | adol | mixed | individual and family-based | high | |
| McLeavey 1994 | ipt | supp | eu | yes | clin | 0.74 | yadult | outpatient | individual | NA | |
| McMain 2017 | dbt | ecau | can | yes | clin | 0.79 | adult | outpatient | group | NA | |
| McManama O'Brien 2018 | other | cau | usa | yes | clin | 0.80 | adol | inpatient | individual and family-based | high | |
| Melvin 2006 | cbt | ecau | au | no | clin | 0.66 | adol | outpatient | individual and family-based | high | |
| Miklowitz 2020 | fam | other ctr | usa | no | clin | 0.65 | adol | outpatient | family-based | high | |
| Monteso-Curto 2015 | pst | cau | eu | no | NA | 0.97 | adult | outpatient | individual | high | |
| Morley 2014 | cbt | cau | au | yes | clin | 0.37 | adult | outpatient | individual and group | high | |
| Morthorst 2012 | pst | cau | eu | yes | oth | 0.76 | adult | outpatient | individual | NA | |
| Naidoo 2014 | other | cau | oth | yes | oth | 0.52 | adult | outpatient | individual | high | |
| Nakimuli-Mpungu 2020 | cbt | other ctr | oth | no | clin | 0.54 | adult | outpatient | group | high | |
| O'Connor 2020 | cbt | cau | usa | yes | clin | 0.45 | adult | inpatient | individual | high | |
| O'Toole 2019 | other | cau | eu | yes | clin | 0.57 | adult | outpatient | individual | high | |
| Pachankis 2022 | cbt | cau | usa | check | com | 0.40 | adult | outpatient | individual | high | |
| Patel 2017 | other | cau | oth | no | clin | 0.77 | adult | outpatient | individual | low | |
| Patsiokas 1985 | cbt | other ctr | usa | yes | clin | NA | adult | inpatient | individual | high | |
| Peters 2010 | cbt | wl | uk | no | clin | 0.15 | adult | outpatient | individual | high | |
| Pfeiffer 2019 | other | cau | usa | yes | clin | 0.53 | adult | outpatient | individual | high | |
| Pigeon 2017 | cbt | other ctr | usa | no | clin | 0.11 | old | outpatient | individual | high | |
| Pigeon 2019 | cbt | cau | usa | yes | clin | 0.20 | adult | outpatient | individual | high | |
| Pineda 2013 | fam | cau | au | yes | clin | 0.75 | adol | outpatient | individual | high | |
| Pistorello 2020 | cams | cau | usa | yes | clin | 0.68 | yadult | outpatient | individual | NA | |
| Power 2003 | cbt | cau | au | yes | clin | NA | yadult | outpatient | individual | high | |
| Pratt 2015 | cbt | cau | uk | yes | oth | 0 | adult | outpatient | individual | high | |
| Rahnama 2016 | img | cau | oth | yes | oth | NA | NA | outpatient | individual | high | |
| Robinson 2024 | cbt | cau | us | no | com | 0.56 | ado | outpatient | group | high | |
| Rossouw 2012 | mbt | cau | uk | yes | clin | 0.85 | adol | outpatient | individual and family-based | NA | |
| Rudd 1996 | other | cau | usa | yes | clin | 0.19 | yadult | outpatient | group | high | |
| Rudd 2015 | cbt | cau | usa | yes | clin | 0.13 | adult | outpatient | individual | high | |
| Ryberg 2019 | cams | ecau | eu | yes | clin | 0.53 | adult | mixed | individual | low | |
| Salkovskis 1990 | pst | cau | uk | yes | clin | 0.50 | adult | outpatient | individual | high | |
| Samaraweera 2007 | cbt | cau | oth | yes | com | 0.60 | adult | outpatient | individual | high | |
| Sanz Cruces 2018 | cbt | wl | eu | no | clin | 0.55 | adult | outpatient | group | high | |
| Sedghy 2022 | cbt | cau | oth | yes | oth | 0.47 | adult | outpatient | individual | high | |
| Shaygan 2022 | other | cau | oth | no | NA | 0.71 | adult | outpatient | group | high | |
| Sheaves 2019 | other | cau | uk | no | clin | 0.42 | adult | outpatient | individual | high | |
| Simpson 2011 | cbt | wl | au | no | clin | NA | adult | outpatient | group | high | |
| Sinniah 2017 | cbt | cau | oth | yes | clin | 0.70 | adult | outpatient | individual | high | |
| Slee 2008 | cbt | cau | eu | yes | clin | 0.94 | adult | outpatient | individual | high | |
| Slesnick 2019 | cbt | cau | usa | yes | clin | 0.41 | yadult | outpatient | individual | high | |
| Spirito 2015 | fam | cau | usa | yes | clin | 0.83 | adol | outpatient | individual and family-based | high | |
| Springer 1996 | cbt | other ctr | usa | no | clin | 0.68 | adult | inpatient | group | high | |
| Tang 2009 | ipt | supp | eas | yes | oth | 0.66 | adol | outpatient | classroom | high | |
| Tarrier 2014 | cbt | cau | uk | yes | clin | 0.37 | adult | outpatient | individual | high | |
| Tighe 2017 | other | wl | au | yes | com | 0.64 | adult | NA | gsh | high | |
| Turner 2000 | dbt | other ctr | usa | yes | oth | 0.79 | yadult | outpatient | individual and group | high | |
| Tyrer 2003 | cbt | cau | uk | yes | oth | 0.68 | adult | outpatient | individual | NA | |
| Van Beek 2013 | cbt | cau | eu | yes | clin | 0.59 | adult | outpatient | group | high | |
| Van der Sande 1997 | other | cau | eu | yes | clin | 0.66 | adult | mixed | individual | NA | |
| Van Orden 2021 | other | cau | usa | yes | clin | 0.68 | old | home | individual | high | |
| Van Wel 2009 | other | ecau | eu | yes | clin | 0.86 | adult | outpatient | group | NA | |
| Verheul 2003 | dbt | cau | eu | no | clin | 1 | adult | outpatient | individual and group | NA | |
| Walker 2016 | dyn | other ctr | uk | yes | oth | 1 | adult | outpatient | individual | high | |
| Waraan 2021 | fam | other | eu | no | clin | 0.87 | adol | outpatient | family-based | high | |
| Ward-Ciesielski 2017 | other | other ctr | usa | yes | clin | 0.36 | adult | outpatient | individual | high | |
| Wei 2013 | cbt | cau | eas | yes | oth | 0.76 | adult | outpatient | individual | high | |
| Weinberg 2006 | cbt | cau | usa | yes | com | 1 | adult | outpatient | individual | high | |
| Weinstein 2018 | fam | cau | usa | no | clin | 0.41 | child | outpatient | individual and family-based | high | |
| Wharff 2019 | fam | cau | usa | yes | oth | 0.72 | adol | inpatient | family-based | high | |
| Wilks 2018 | dbt | wl | usa | yes | com | 0.69 | adult | NA | individual | high | |
| Wood 2001 | other | cau | uk | yes | clin | 0.78 | adol | outpatient | group | high | |
| Wu (R) 2021 | other | wl | eas | yes | com | 0.77 | yadult | outpatient | individual and group | high | |
| Wu 2023 | dbt | other | eas | yes | clin | NA | adult | outpatient | other | high | |
| Xie 2023 | other | ecau | eas | no | clin | 0.40 | adult | outpatient | other | NA | |
| Yen 2019 | other | ecau | usa | yes | clin | 0.69 | adol | inpatient | individual and family-based | high | |
| Zhao 2023 | img | wl | eas | no | clin | 0.84 | adult | outpatient | individual | High | |
| Note. Assip: Attempted Suicide Short Intervention Program. Cams: Collaborative Assessment & Management of Suicidality. Cbt: Cognitive Behavioural Therapy. Dbt: Dialectical Behavioural Therapy. Dyn: Psychodynamic Therapy. Fam: Family-based Therapy. Img: EMDR and imagery based therapies. Ipt: Interpersonal Therapy. Mbct: Mindfulness based Cognitive Therapy. Mbt: Mentalisation-based Therapy. Mea: Meaning making Therapy. Pst: Problem-solving Therapy. Spi: Safety-planning or related interventions. Cau: Care as usual. Ecau: Enhanced Care as usual. Supp: Supportive counselling. Wl: Wait-list. AU: Australia or New Zealand. CAN: Canada. EAS: East Asia. EU: European Union. UK: United Kingdom. USA: United States of America. Suic incl: Suicidality as inclusion criterion. Com: Community. Clin: Clinical. Oth: Other recruitment. Adol: Adolescent, Yadult: young adult. | | | | | | | | | | |  |

## Appendix D: Forest plots per format

Individual treatment - Ideation


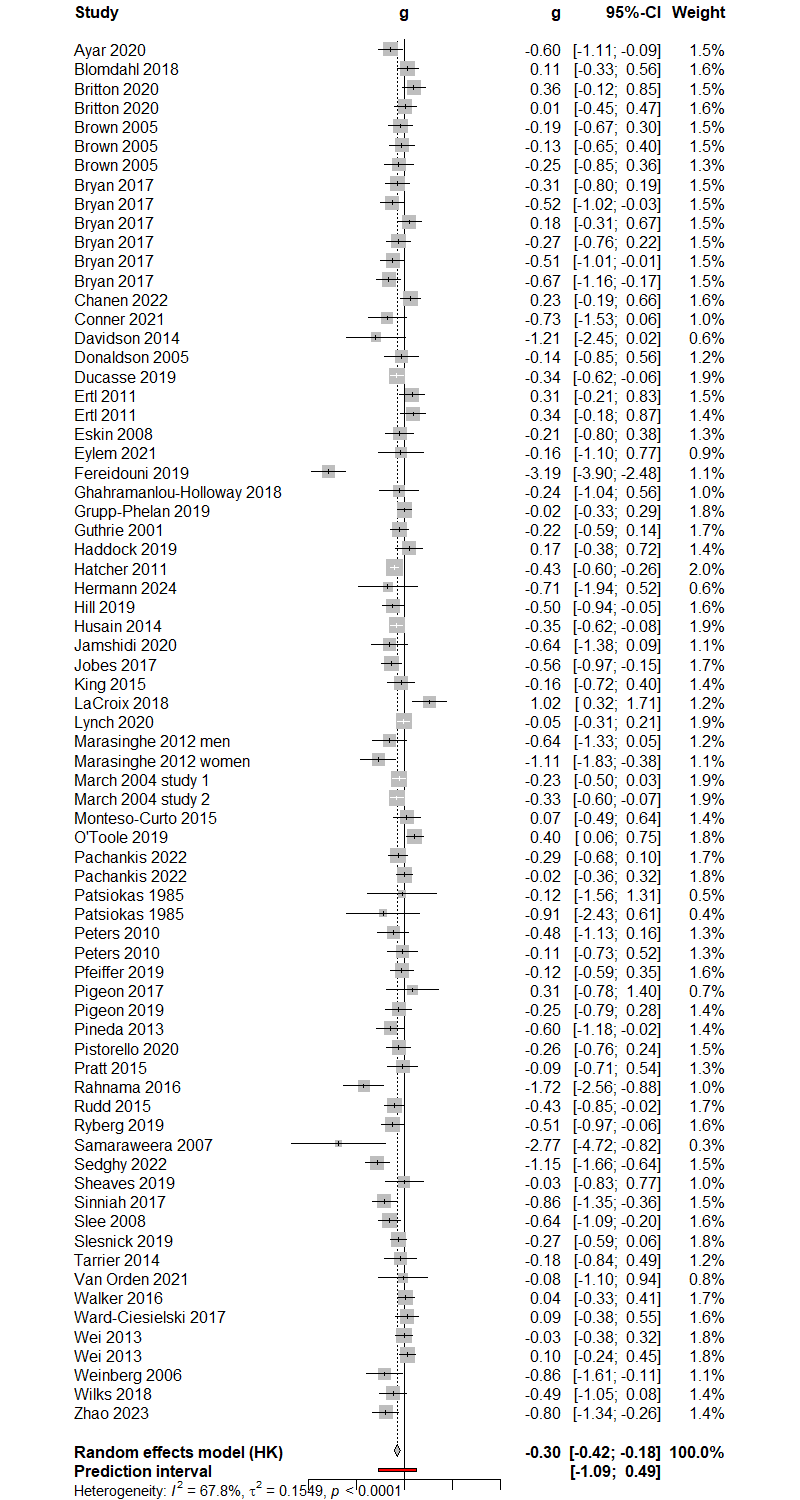


Individual treatment – Attempts


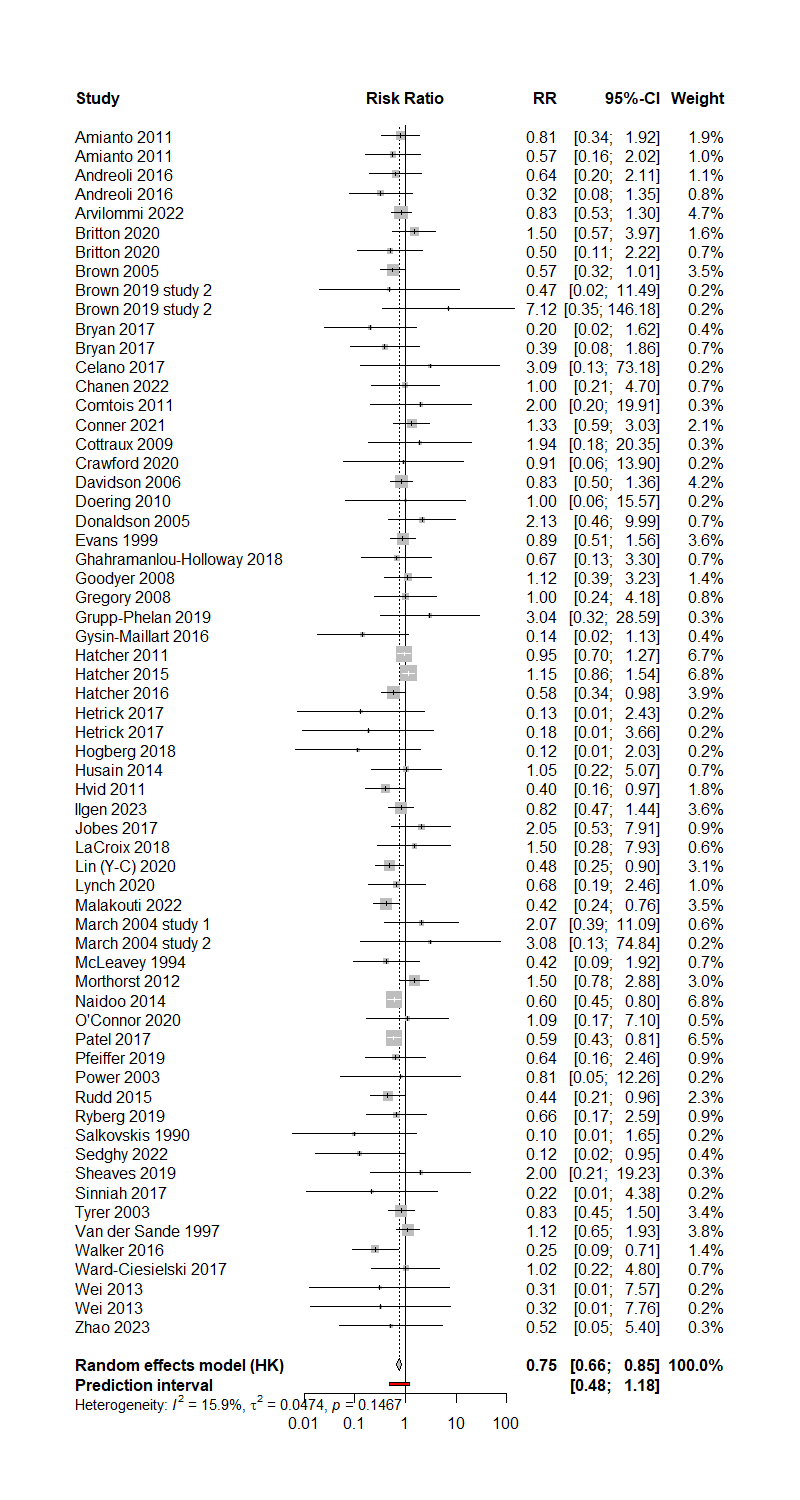


Group treatment – Ideation


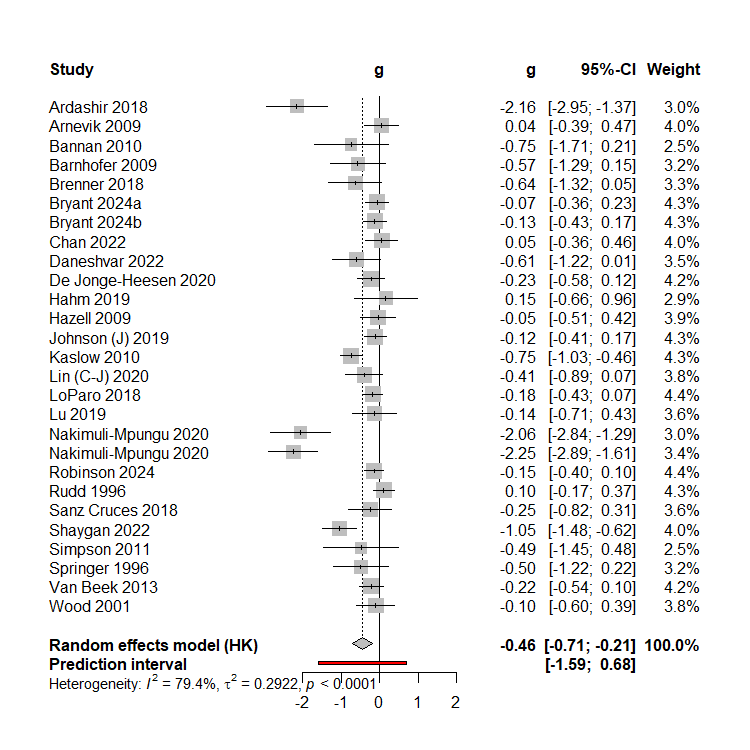


Group treatment – Attempts


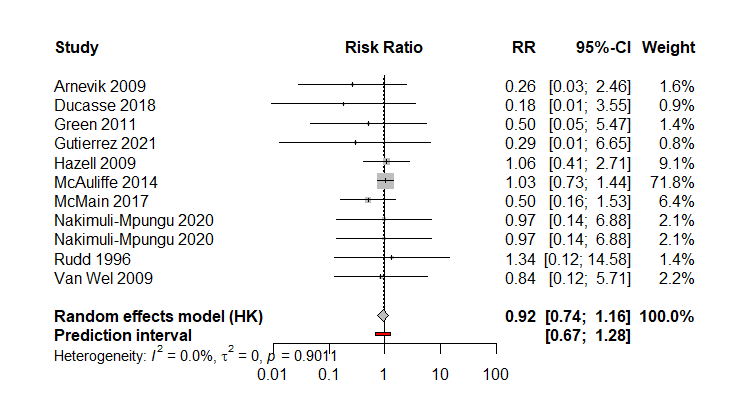


Family-based treatment – Ideation


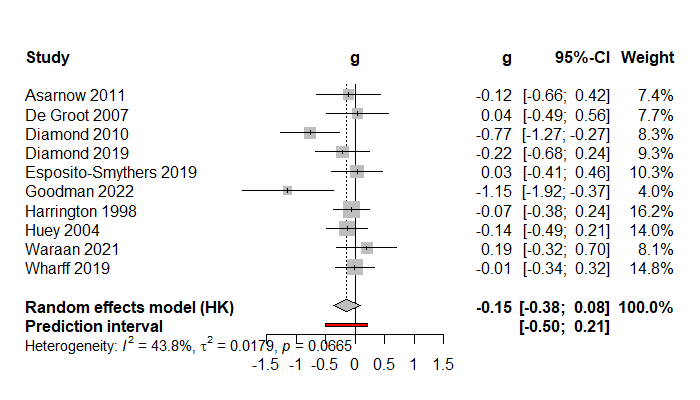


Family-based treatment – Attempts


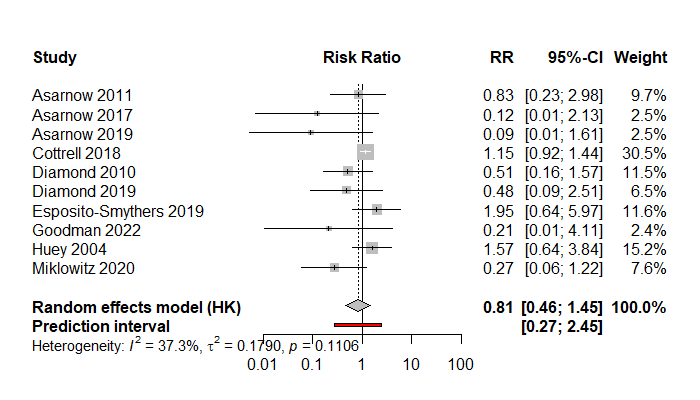


Individual and Group treatment – Ideation


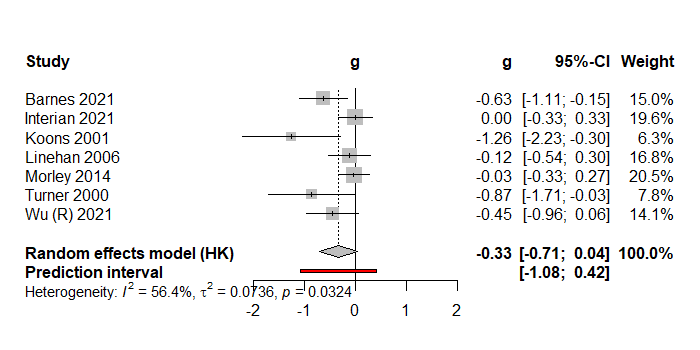


Individual and Group treatment – Attempts


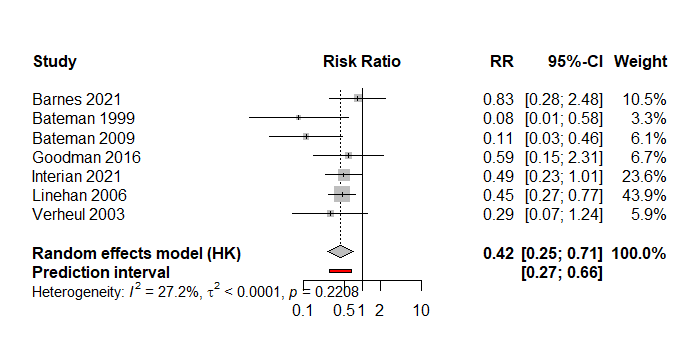


Individual and Family-based treatment – Ideation


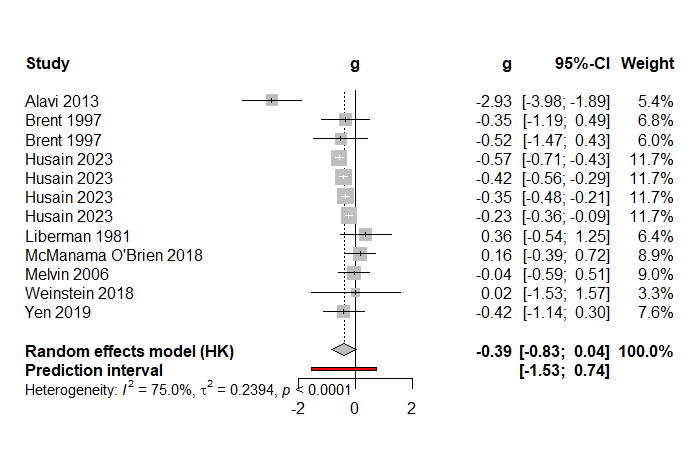


Individual and Family-based treatment – Attempts


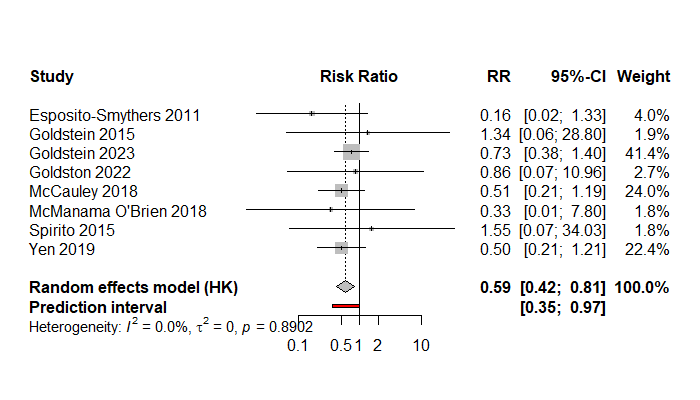


## Appendix E: R code

# Code for meta analysis on therapy format - Date: February 2025

## Setting up

#Only run this the first time
# install.packages("remotes")
# remotes::install_github("metapsy-project/metapsyTools")
# install.packages("tidyverse")
# install.packages("readxl")
# install.packages("rmeta")
# remotes::install_github("MathiasHarrer/dmetar")

Load the packages

library(tidyverse)

library(metapsyTools)

library(meta)

library(metafor)

library(rmeta)
library(readxl)
library(dmetar)

Load the data

data <- read_excel("/file name removed")

## Prepping the data

# choose proper control groups
table(data$condition_arm2, useNA = "always")

target <- c("wl", "cau", "ecau", "mixed", "other", "other ctr", "supp", "placebo")
data %>% filter(condition_arm2 %in% target) -> data

# Now we make a dataset for analysing ideation as outcome
data %>%
 mutate(across (c(time_weeks, mean_arm1, mean_arm2, sd_arm1, sd_arm2, n_arm1, n_arm2,
 si_event_arm1, si_event_arm2, si_n_arm1, si_n_arm2, mean_change_arm1,
 mean_change_arm2, sd_change_arm1, sd_change_arm2, n_change_arm1,
 n_change_arm2, precalc_g, precalc_g_se), as.numeric)) %>%
 filter (mean_arm1 > 0 | mean_change_arm1 > 0 | si_n_arm1 > 0 | precalc_g > 0 | precalc_g < 0) %>%
 select (study, condition_arm1, condition_arm2, multi_arm1, multi_arm2, instrument, country,
 dsm_icd, disorder_suic, age_group, format, recruitment, time_weeks,
 mean_arm1, mean_arm2, sd_arm1, sd_arm2, n_arm1, n_arm2,
 si_event_arm1, si_event_arm2, si_n_arm1, si_n_arm2, mean_change_arm1, mean_change_arm2,
 sd_change_arm1, sd_change_arm2, n_change_arm1, n_change_arm2, precalc_g, precalc_g_se, rob_total) %>%
 rename (event_arm1 = si_event_arm1,
 event_arm2 = si_event_arm2,
 totaln_arm1 = si_n_arm1,
 totaln_arm2 = si_n_arm2,
 rob = rob_total) -> ideation

ideation %>% filter (mean_arm1 > 0 | precalc_g > 0 | precalc_g < 0) -> ideation_cont
ideation_cont$outcome_type <- "msd"

ideation %>% filter (mean_change_arm1 > 0) -> ideation_change
ideation_change$outcome_type <- "change"

ideation %>% filter (totaln_arm1 > 0) -> ideation_dich
ideation_dich$outcome_type <- "deterioration"

# Sign of g based on binary outcomes is flipped!
ideation_dich$event_arm1 <- ideation_dich$totaln_arm1 - ideation_dich$event_arm1
ideation_dich$event_arm2 <- ideation_dich$totaln_arm2 - ideation_dich$event_arm2

ideation_cont %>% bind_rows(ideation_change) %>% bind_rows(ideation_dich) -> ideation

# for analysis of attempts
data %>%
 mutate(across (c(time_weeks, mean_arm1, mean_arm2, sd_arm1, sd_arm2, n_arm1, n_arm2,
 event_arm1, event_arm2, totaln_arm1, totaln_arm2), as.numeric)) %>%
 filter(event_arm1 > 0 | event_arm2 > 0) %>%
 select(study, format, condition_arm1, condition_arm2, multi_arm1, multi_arm2, instrument, country,
 dsm_icd, disorder_suic, age_group, recruitment, time_weeks, event_arm1, event_arm2, totaln_arm1, totaln_arm2, rob_total) %>%
 rename(rob = rob_total) -> attempts

attempts$outcome_type <- "RR"

# check with MetaPsyTools
ideation <- checkDataFormat(ideation)

checkConflicts(ideation)

ideation$time <- "post"
ideation$rating <- "NA"

ideation <- calculateEffectSizes(ideation)

#Do the same for attempts
attempts <- checkDataFormat(attempts)

checkConflicts(attempts)

attempts$time <- "post"
attempts$rating <- "NA"
attempts <- calculateEffectSizes(attempts)

## Run meta-analyses

One for each type of format that has enough

table(ideation$format)

##
## classroom fam grp gsh ind ind and fam
## 1 10 31 1 72 12
## ind and grp other
## 7 1

table(attempts$format)

##
## fam grp ind ind and fam ind and grp other
## 10 11 63 8 7 1

Individual - ideation

ideation_ind <- ideation %>%
 filter(format == "ind")
res_ideation_ind <- runMetaAnalysis(ideation_ind)

Individual - attempts

attempts_ind <- attempts %>%
 filter(format == "ind")
res_attempts_ind <- runMetaAnalysis(attempts_ind, es.measure = "RR")

Group - ideation

ideation_grp <- ideation %>%
 filter(format == "grp")
res_ideation_grp <- runMetaAnalysis(ideation_grp)

attempts_grp <- attempts %>%
 filter(format == "grp")
res_attempts_grp <- runMetaAnalysis(attempts_grp, es.measure = "RR")

Family - ideation

ideation_fam <- ideation %>%
 filter(format == "fam")
res_ideation_fam <- runMetaAnalysis(ideation_fam)

Family - attempts

attempts_fam <- attempts %>%
 filter(format == "fam")
res_attempts_fam <- runMetaAnalysis(attempts_fam, es.measure = "RR")

Individual and Group - ideation

ideation_indgrp <- ideation %>%
 filter(format == "ind and grp")
res_ideation_indgrp <- runMetaAnalysis(ideation_indgrp)

Individual and Group - attempts

attempts_indgrp <- attempts %>%
 filter(format == "ind and grp")
res_attempts_indgrp <- runMetaAnalysis(attempts_indgrp, es.measure = "RR")

Individual and Family centred - ideation

ideation_indfam <- ideation %>%
 filter(format == "ind and fam")
res_ideation_indfam <- runMetaAnalysis(ideation_indfam)

Individual and Family centred - attempts

attempts_indfam <- attempts %>%
 filter(format == "ind and fam")
res_attempts_indfam <- runMetaAnalysis(attempts_indfam, es.measure = "RR")

## Subgroup analysis

For ideation

ideation_format <- ideation %>%
 filter(!format %in% c("classroom", "gsh", "other"))

res_ideation_format <- runMetaAnalysis(ideation_format)

sg_i <- subgroupAnalysis(res_ideation_format, format, .which.run = ("threelevel.che"))

## - [OK] 'model.threelevel.che' used for subgroup analyses.

sg_i

For attempts

attempts_format <- attempts %>%
 filter(!format %in% c("other"))

res_attempts_format <- runMetaAnalysis(attempts_format, es.measure = "RR")

sg <- subgroupAnalysis(res_attempts_format, format, .which.run = ("threelevel.che"))

### Check final data

Get new dataset to make the summary table

merged <- data %>%
 mutate(in_ideation_dataset = ifelse(study %in% ideation_format$study, 1, 0))
merged <- merged %>%
 mutate(in_attempts_dataset = ifelse(study %in% attempts_format$study, 1, 0))

Download files to new datasets to do analyses in jamovi

write.csv(merged, "/alle papers.csv")
write.csv(ideation_format, "/ideation MA.csv")
write.csv(attempts_format, “/attempts MA.csv")

### Forest plots for appendices

plot(res_ideation_ind) #width 800, height 1300

plot(res_attempts_ind) #width 800, height 1300

plot(res_ideation_grp) #width 700, height 700

plot(res_attempts_grp) #width 600, height 400

plot(res_ideation_fam) #width 650, height 400

plot(res_attempts_fam) #width 650, height 400

plot(res_ideation_indgrp) #width 600, height 350

plot(res_attempts_indgrp) #width 650, height 350

plot(res_ideation_indfam) #width 650, height 350

plot(res_attempts_indfam) #width 650, height 350

### Publication bias

For ideation

funnel(res_ideation_format$model.threelevel.che)

eggers.test(res_ideation_format$model.overall)

For attempts

funnel(res_attempts_format$model.threelevel.che)

eggers.test(res_attempts_format$model.overall)
